# Markers in Infants of Mothers With Asthma and Associations With Respiratory Outcomes

**DOI:** 10.1111/all.70044

**Published:** 2025-09-15

**Authors:** Carla Rebeca Da Silva Sena, Gabriela Martins Costa Gomes, Noëmi Künstle, Olga Gorlanova, Andrea Marten, Sven Schulzke, Florian Wyler, Vanessa E. Murphy, Paul D. Robinson, Peter D. Sly, Jakob Usemann, Benjamin Stoecklin, Ruth Steinberg, Sophie Yammine, Loretta Müller, Philipp Latzin, Pablo Sinues, Peter G. Gibson, Joerg Mattes, Adam Collison, Urs Frey, the BILD and BLT Study Groups

**Affiliations:** ^1^ University Children's Hospital Basel UKBB, University of Basel Basel Switzerland; ^2^ Asthma & Breathing Research Program, Hunter Medical Research Institute Newcastle New South Wales Australia; ^3^ University of Newcastle Newcastle New South Wales Australia; ^4^ Division of Pediatric Respiratory Medicine and Allergology, Department of Pediatrics Inselspital, Bern University Hospital, University of Bern Bern Switzerland; ^5^ Department of Respiratory Medicine Queensland Children's Hospital Brisbane Queensland Australia; ^6^ Children's Health and Environment Program, Child Health Research Centre The University of Queensland Brisbane Queensland Australia; ^7^ Lung Precision Medicine (LPM), Department for Biomedical Research (DBMR) University of Bern Bern Switzerland; ^8^ Department of Biomedical Engineering University of Basel Allschwil Switzerland; ^9^ Respiratory & Sleep Medicine Department John Hunter Hospital Newcastle New South Wales Australia; ^10^ School of Medicine and Public Health, College of Health, Medicine and Wellbeing University of Newcastle Newcastle New South Wales Australia; ^11^ Paediatric Respiratory & Sleep Medicine Department John Hunter Children's Hospital Newcastle New South Wales Australia

**Keywords:** asthma, asthma during pregnancy, bronchiolitis, infant lung function, proteins

## Abstract

**Background:**

In utero mechanisms related to oxidative stress response, inflammation, and extracellular matrix turnover may influence fetal lung development in the offspring of asthmatic mothers. Therefore, we aimed to determine whether levels of cord blood proteins differ between the offspring of asthmatic and nonasthmatic mothers. In addition, we aimed to examine if these proteins are associated with infant lung function, bronchiolitis hospitalization in infancy, and asthma at six years.

**Methods:**

We compared protein levels of infants (*n* = 715) from the Swiss Basel‐Bern Infant Lung Development and the Australian Breathing for Life Trial birth cohorts using Tobit regression and network analyses. Using adjusted linear and logistic regression, we determined their association with postnatal infant lung function, bronchiolitis hospitalization in infancy, and asthma at six years.

**Results:**

Infants born to asthmatic mothers had lower levels of matrix metalloproteinase‐9 (MMP‐9, *β*‐coefficient [*β*] −0.67, 95% confidence interval [−1.07; −0.27] *p*_adj_ = 0.009) and Interferon gamma (IFN‐γ, *β* −0.77 [−1.21; −0.32], *p*_adj_ = 0.009), and higher levels of p62 (*β* 1.15 [0.30; 2.00], *p*_adj_ = 0.030). p62 levels were inversely associated with minute ventilation (*β* −16.18 [−28.44; −3.91], *p*_adj_ = 0.032). Functional residual capacity values were inversely associated with both IFN‐γ (*β* −1.26, [−2.41; −0.11], *p*_adj_ = 0.063) and MMP‐9 levels (*β* −1.27, [−2.53; −0.01], *p*_adj_ = 0.063). MMP‐9 levels were inversely associated with both the risk of bronchiolitis hospitalization (odds ratio 0.47, [0.29; 0.77], *p*_adj_ = 0.004) and the risk of asthma (aOR 0.53, 95% CI, 0.32–0.86, *p*_adj_ = 0.033).

**Conclusions:**

Protein levels differed between offspring of asthmatic and non‐asthmatic mothers. These markers were associated with postnatal lung function, bronchiolitis hospitalization, and asthma.

## Graphical abstract

In the BILD and BLT cohorts, we compared cord blood proteins in infants of mothers with and without asthma during pregnancy. Groups showed different protein levels. These markers were associated with postnatal lung function, bronchiolitis hospitalization, and asthma. Additionally, p62 was inversely associated with minute ventilation, MMP‐9 inversely with FRC and bronchiolitis/asthma risk, while IFN‐γ and MMP‐9 was positively associated with lung clearance index, highlighting distinct protein–lung function and disease associations. Abbreviations: BILD, Basel‐Bern Infant Lung Development cohort; BLT, Breathing for Life cohort; FRC, functional residual capacity; IFN‐γ, interferon gamma; p62, ubiquitin‐binding protein sequestosome 1; MMP‐9, matrix metalloproteinase‐9.

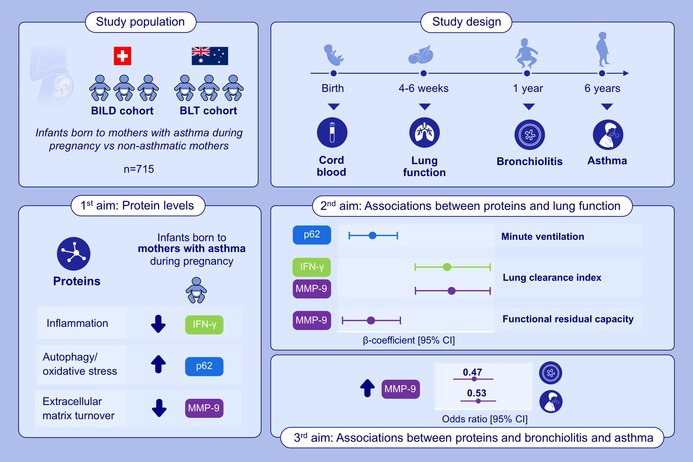

## 1 Introduction

Respiratory morbidity in childhood and adulthood is a global and increasing problem, with multiple etiologies [1]. One known contributor is asthma symptoms during pregnancy, which affects up to 17% of pregnancies globally [2]. We and others have previously demonstrated that children born to mothers with asthma during pregnancy have reduced lung function at age 5–6 weeks [3] and a higher risk of developing respiratory morbidities in childhood [4, 5, 6]. Additionally, we have shown that environmental factors during pregnancy affect the cord blood immune cell profiles [7]. These changes are linked to reduced lung function [8] and an increased risk of bronchiolitis hospitalization in the first year of life [9].

It has been shown that the immune system during pregnancy mimics a stress response, with a shift towards a type 2 (Th2)‐dominant profile influencing pregnancy outcomes and susceptibility to immune‐mediated diseases [10]. Several proteins are involved in the developing immune system of the respiratory tract in utero [11], and also play a role in lung growth and development in utero, such as lung branching morphogenesis (EGF, VEGF‐A) [12], alveolar formation regulating epithelial and mesenchymal cell proliferation and differentiation (PDGF‐AA, TGF‐β) [13], regulation of the extracellular matrix components (MMP‐9) [14], regulation of inflammation (IL‐1β, IL‐8, TNF‐α, IFN‐γIL‐4, IL‐13, Il‐17), and cellular homeostasis, cellular aging, and autophagy (ATG5, Beclin‐1, SIRT1, p62) [15].

The mechanisms underlying lung function deficits and respiratory morbidity are not fully understood, but preclinical evidence suggests that maternal asthma alters the in utero environment, which affects fetal lung structural, functional, and immune development [16, 17, 18, 19, 20]. Specific proteins in cord blood related to inflammation, autophagy/oxidative stress response, and extracellular matrix turnover (ECM‐turnover) [21] may give indirect evidence of alterations in the in utero environment, which potentially lead to lung functional growth abnormalities and respiratory morbidity in the offspring.

Given the differences in postnatal lung function [3] and respiratory morbidity [2, 22] between infants born to mothers with asthma during pregnancy and those born to non‐asthmatic mothers, we hypothesize that the in utero mechanisms related to autophagy/oxidative stress response, inflammation, and ECM turnover may have an effect on fetal lung organogenesis, and subsequently on postnatal lung function and respiratory morbidity in the offspring.

Therefore, in cord blood collected from the Australian Breathing for Life Trial (BLT) [23] and the Swiss Basel–Bern Infant Lung Development (BILD) [24] prospective birth cohorts, we firstly aimed to identify differences in the specific protein markers between infants of mothers with asthma during pregnancy and those of non‐asthmatic mothers. Secondly, we aimed to determine whether these representative differing cord blood protein markers were associated with postnatal lung function at 4–6 weeks of age (tidal breathing parameters, lung volume, and ventilation inhomogeneity), with bronchiolitis hospitalization in the first year of life, and with asthma at six years of age.

## 2 Methods

### 2.1 Study Design

The study was designed with two aims. First, we identified a selected fingerprint of cord blood protein markers, presented in three “mechanistic” categories: “inflammation,” “autophagy/oxidative stress,” and “ECM‐turnover” (Table S1). Then we compared protein levels from infants born to asthmatic mothers during pregnancy (defined as: self‐reported, doctor‐diagnosed, asthma) to infants of non‐asthmatic mothers. Subsequently, we identified the most distinctive and relevant representative protein marker for each category. In the second aim, we determined whether these three representative proteins were associated with lung function at the age of 4–6 weeks (second aim, part one) and with bronchiolitis hospitalization in the first year of life (second aim, part two), and asthma at six years of age (second aim, part three).

The BLT [23] and BILD [24] cohorts are described in the Supporting Information [3]. For both cohorts, written informed parental consent was obtained at the time of enrollment. Both studies are approved by the local Human Ethics Committees, and informed consent was obtained for all participants included.

### 2.2 Outcomes: Cord Blood Protein Levels (First Aim)

Immediately after birth, umbilical cord blood was collected in EDTA tubes, then centrifuged to obtain plasma and stored at −80°C until analysis. Plasma proteins were analyzed by Enzyme‐Linked Immunosorbent Assay (ELISA) or Luminex (Further details are described in the Supporting Information). Due to limited sample volume, some patients had only a subset of proteins measured, while others had all proteins assessed.

The inflammation‐related markers were interleukin (IL)‐1 beta (IL‐1β), IL‐8, tumor necrosis factor alpha (TNF‐α), interferon gamma (IFN‐γ), IL‐4, IL‐13, and IL‐17A. The autophagy‐related markers were ubiquitin‐binding protein sequestosome 1 (p62/SQSTM1, referred to hereafter as p62), beclin‐1 (Beclin‐1), sirtuin 1 (SIRT1), and autophagy protein 5 (ATG5). The ECM‐turnover‐related markers were matrix metalloproteinase‐9 (MMP‐9), transforming growth factor beta‐1 (TGF‐ß1), platelet‐derived growth factor AA (PDGF‐AA), epidermal growth factor (EGF) and vascular endothelial growth factor A (VEGF‐A).

### 2.3 Infant Lung Function at 4–6 Weeks of Age (Second Aim, Part One)

Infant lung function testing was conducted according to the European Respiratory Society/American Thoracic Society standards [25, 26]. Equipment, collection, and lung function analysis were methodologically aligned and synchronously performed between the two cohorts (see Supporting Information) [27].

Tidal breathing parameters included minute ventilation and the ratio of total time to peak tidal expiratory flow (tPTEF) as a percentage of total expiratory time (tE) (tPTEF/tE%). Lung volume measured by functional residual capacity (FRC) and lung clearance index (LCI) was calculated using an improved algorithm to analyze molar mass (MM)‐based infant sulfur hexafluoride multiple breath washout (SF_6_ MBW) measurements [27].

*Bronchiolitis hospitalization (second aim, part two)* was identified in the electronic medical records for both cohorts.

*Asthma in childhood (second aim, part three)* was assessed during follow‐up assessments conducted at six years of age where parents were asked to report doctor‐diagnosed asthma.

### 2.4 Statistical Analysis

Study characteristics are presented as frequencies and percentages for categorical variables, and as means with standard deviations (SD) or medians with interquartile ranges (IQR) for continuous variables. Protein data were log2‐transformed to achieve normality [28]. Throughout this manuscript, infants born to mothers with asthma during pregnancy are referred to as “born to asthmatics” and negative cases as “born to non‐asthmatics”. All models and adjustments are described in the Supporting Information.

We applied two complementary approaches to assess differences in cord blood protein markers between infants born to mothers with and without asthma during pregnancy (1). Protein levels were compared using Tobit regression (a method suitable for censored data where some protein concentrations fall below the assay's detection limit accounting) [29, 30], where maternal asthma during pregnancy was treated as the primary exposure in the model (2). To identify interconnected proteins among the group of infants born to asthmatics and non‐asthmatics, we explored correlation network structures using centrality parameters to identify the most influential nodes. Spearman's correlation was used to calculate protein relationships, with edges included for absolute correlation if *r* ≥ 0.3, to preclude exclusion in network connectivity. Node centrality parameters included: (i) *degree centrality* (number of connections a node has), (ii) *betweenness centrality* (frequency of a node acting as a bridge), (iii) *strength centrality* (the proximity magnitude of connection between nodes). Both methods pointed to consistent proteins of interest (IFN‐γ, MMP‐9, and p62), which were subsequently prioritized for further investigation for the second aim [31].

Using linear regression analysis, we tested the association between the protein levels (exposure) and postnatal lung function measurements (outcome), adjusting for covariates. Using logistic regression, we calculated bronchiolitis hospitalization and asthma in childhood risk with protein levels as the exposure. Results are expressed as adjusted odds ratios (aOR) with 95% confidence intervals (95% CI), adjusted for covariates. Covariates were selected on the basis of our previous studies [3, 8, 32, 33, 34, 35, 36, 37] and included sex, having siblings at birth, gestational age, maternal smoking during pregnancy, mode of delivery (vaginal or cesarean section), birth weight (*z*‐score), study center, and time to processing sample in days, weight at visit (in g), breastfeeding up to visit day, and season of birth. Additionally, we calculated variance inflation factors (VIFs) after each regression to assess multicollinearity among predictors.

### 2.5 Sensitivity Analysis

To assess the robustness of our models, we conducted a sensitivity analysis including only children born via vaginal delivery, excluding those born by Cesarean section. Additionally, we performed LASSO regression adjusting for variables identified as relevant by the method.

*p*‐values were adjusted for multiple testing using the Benjamini–Hochberg method, accounting for the number of proteins for each aim. *p*‐values and *p*_adj_‐values < 0.05 were considered significant. Data analysis was performed in Stata 16 (StataCorp, College Station, TX), and R version 4.3.2 (R Foundation, Vienna, Austria).

## 3 Results

By merging the BILD and BLT cohorts (Table S2, Figure S1) a total of 715 term infants had cord blood collected at birth; *n* = 135 were born to asthmatics (103 BLT, 32 BILD) and *n* = 580 to non‐asthmatics (BILD). The groups' demographic characteristics differed regarding mode of delivery (more commonly Cesarean section in the born to asthmatics group, 32% vs. 22%, *p* = 0.009), gestational age (39.7 weeks vs. 39.4, *p* = 0.01), birth length (51.1 cm vs. 49.9 cm, *p* > 0.001), and weight in z‐score (0.10 vs. −0.14, *p* = 0.001, Table 1). In addition, infants born to asthmatic mothers had lower l tPTEF/tE% (32.4 vs. 36.0, *p* = 0.001), but higher minute ventilation (1493.7 vs. 1429.7, *p* = 0.022) and LCI (6.9 vs. 6.7, *p* = 0.013) compared to infants born to non‐asthmatic mothers. Table S3 shows the distribution of proteins with level of detection, including raw and log2 transformed data.

**TABLE 1:** Demographic data of all infants and comparison of infants by grouping: infants born to asthmatic mothers versus infants born to non‐asthmatic mothers.

	Total (*n* = 715)	Infants born to asthmatic mothers, *n* = 135	Infants born to non‐asthmatic mothers, *n* = 580	*p*
	BILD *n* = 612, BLT *n* = 103	BILD *n* = 32, BLT *n* = 103	All from BILD	
Maternal baseline characteristics				
Maternal smoking during pregnancy *n* (%)	54 (7.6%)	15 (11.1%)	39 (6.7%)	0.102
Infant baseline characteristics				
First born *n* (%)	333 (46.6%)	69 (51.5%)	264 (45.5%)	0.474
Male *n* (%)	366 (51.2%)	68 (50.4%)	298 (51.4%)	0.849
Delivery type				
Vaginal *n* (%)	553 (78.0%)	90 (67.7%)	463 (80.4%)	**0.002**
Cesarean section *n* (%)	156 (22.0%)	43 (32.3%)	113 (19.6%)	
Season of birth				
Winter *n* (%)	125 (17.8%)	37 (27.4%)	88 (15.2%)	**0.009**
Spring *n* (%)	220 (30.8%)	41 (30.4%)	179 (30.9%)	
Summer *n* (%)	170 (23.8%)	27 (20.0%)	143 (24.7%)	
Fall *n* (%)	200 (28.0%)	30 (22.2%)	170 (29.3%)	
Gestational age in weeks†	39.7 (1.14)	39.4 (1.24)	39.8 (1.11)	**0.001**
Weight at birth, g	3400 (450)	3460 (500)	3390 (440)	0.071
Weight at birth, *z*‐score	−0.14 (0.89)	0.10 (0.98)	−0.20 (0.86)	**0.001**
Length at birth, cm	49.9 (2.3)	51.1 (2.8)	49.7 (2.1)	**< 0.001**
tPTEF/tE% (*n* = 548)	36.0 (10.8)	32.4 (9.7)	36.6 (10.9)	**0.001**
Minute ventilation (*n* = 548)	1429.7 (267.2)	1493.7 (299.0)	1417.9 (260.4)	**0.022**
FRC (*n* = 436)	87.4 (13.3)	89.4 (14.9)	87.5 (12.6)	0.751
LCI (*n* = 436)	6.7 (0.6)	6.9 (0.6)	6.7 (0.6)	**0.013**

*Note:* Categoric variables are presented as counts and percentages (*n* %); Continuous variables are presented as means with SDs and † and as median with IQR. Groups were compared using either a *t*‐test or a chi‐square, as appropriate. *p*‐values < 0.05 are shown in bold.

**FIGURE 1:**
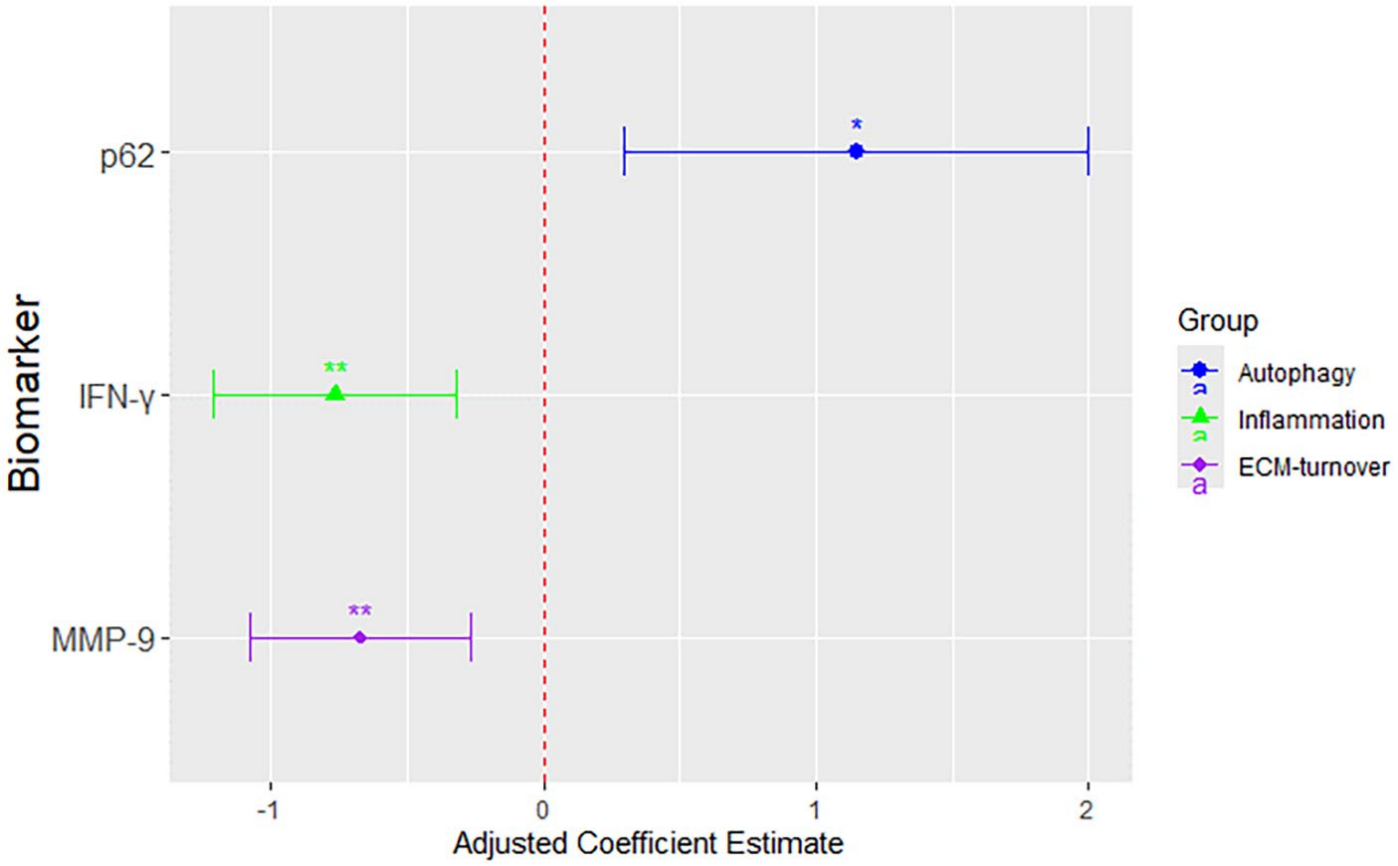
Cord blood protein levels in infants born to a mother with asthma during pregnancy. Beclin‐1, Beclin‐1; ECM‐turnover, extracellular matrix turnover; IFN‐γ, Interferon gamma; MMP‐9, matrix metalloproteinase 9; p62, ubiquitin‐binding protein sequestosome 1. Estimates are reported as a *β*‐coefficient and represent results from a Tobit regression model, where maternal asthma during pregnancy is the primary exposure. Estimates are reported as coefficients with 95% CI log2‐transformed protein levels, also adjusted for sex, having siblings at birth, gestational age, maternal smoking during pregnancy, mode of delivery, birth weight in *z*‐score, study center, and time of cord blood processing. *p*_adj_‐value * < 0.05; **p_adj_‐value < 0.01.

First, we analyzed the differences of protein levels between infants born to asthmatics and those born to non‐asthmatics using univariable analyses (Figure S2). Then we applied Tobit regression adjusting for covariates, which revealed significantly lower IFN‐γ (*β*‐coefficient [*β*] −0.77, 95% CI, −1.21 to −0.32, *p*_adj_ = 0.009), lower MMP‐9 (*β* −0.67, 95% CI, −1.07 to −0.27, *p*_adj_ = 0.009), lower Beclin‐1 (*β* −0.31, 95% CI, −0.53 to −0.10, *p*_adj_ = 0.024), and higher p62 (*β* 1.15, 95% CI, 0.30–2.00, *p*_adj_ = 0.030) in the group of infants born to asthmatics, showing differences in protein levels (Figure 1, Table S4). The full results of the Tobit regression models, including all adjustments, are provided in Tables S10–S12.

**FIGURE 2:**
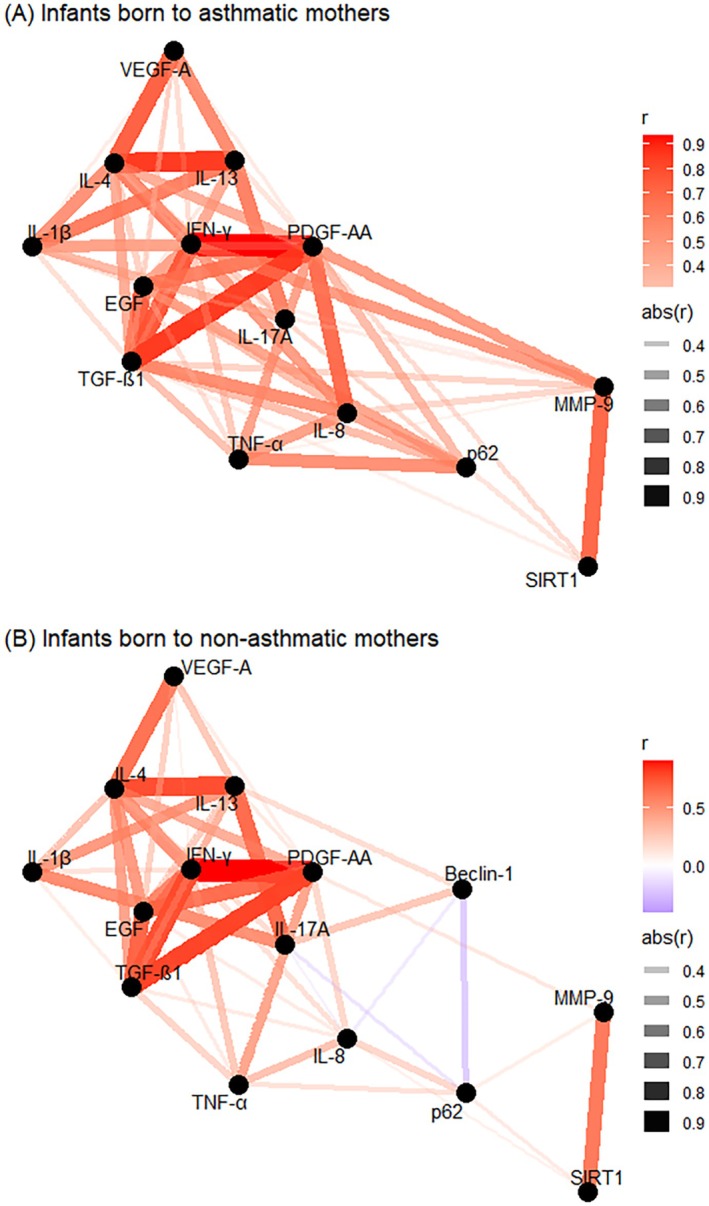
Correlation network in infants born to asthmatic mothers (A) and infants born to non‐asthmatic mothers (B). ATG5, autophagy protein 5; Beclin‐1, Beclin‐1; EGF, Epidermal Growth Factor; IFN‐γ, Interferon gamma; IL‐13, interleukin 13; IL‐17A, interleukin 17A; IL‐1β, interleukin 1β; IL‐4, interleukin 4; IL‐8, interleukin 8; MMP‐9, matrix metalloproteinase 9; p62, ubiquitin‐binding protein sequestosome 1; PDGF‐AA, platelet‐derived growth factor AA; SIRT1, Sirtuin 1; TGF‐β1, transforming growth factor β1; TNFα, tumor necrosis factor α; VEGF‐A, Vascular endothelial growth factor A. Measures of centrality are shown as connections (edges) are established using Spearman's correlation coefficient. Correlations are displayed when the absolute correlation coefficient is > 0.3. Each protein is represented by a node, and edge colors reflect the strength of correlation between proteins.

**FIGURE 3:**
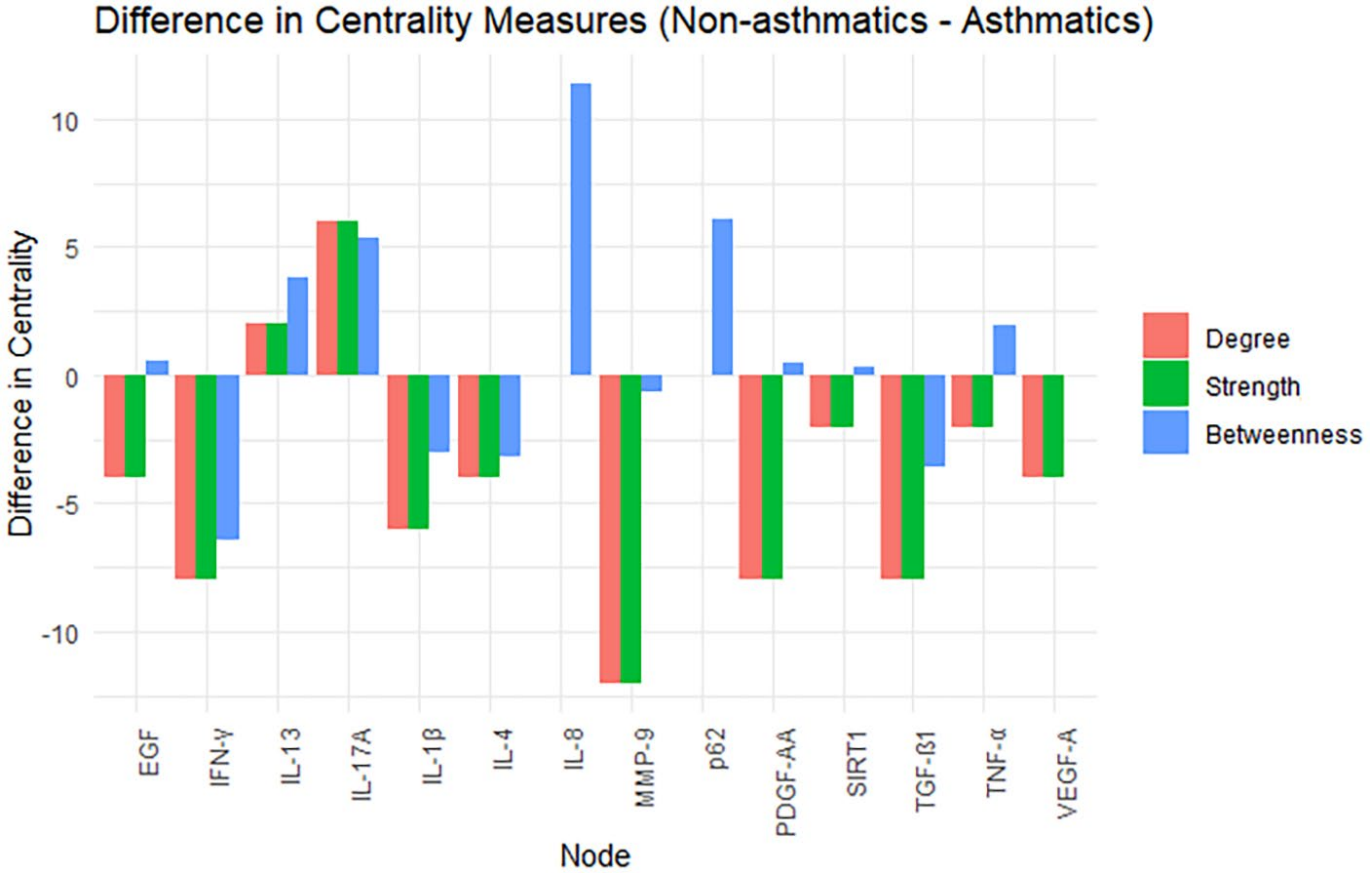
Difference in measures of centrality according to protein markers for infants born to asthmatics and infants born to non‐asthmatics. ATG5, autophagy protein 5; Beclin‐1, Beclin‐1; EGF, epidermal growth factor; IFN‐γ, interferon gamma; IL‐13, interleukin 13; IL‐17A, interleukin 17A; IL‐1β, interleukin 1β; IL‐4, interleukin 4; IL‐8, interleukin 8; MMP‐9, matrix metalloproteinase 9; p62, ubiquitin‐binding protein sequestosome 1; PDGF‐AA, platelet‐derived growth factor AA; SIRT1, Sirtuin 1; TGF‐β1, transforming growth factor β1; TNFα, tumor necrosis factor α; VEGF‐A, vascular endothelial growth factor A. Graph shows network analysis centrality measures including, in red showing the *degree centrality* (which is the number of connections a node has), in green the strength which refers to the magnitude of connection between nodes, and in blue the *betweenness centrality* representing the frequency of a node acting as a bridge.

Next, we performed a correlation network analysis in a subgroup of infants who had all proteins measured, consisting of *n* = 81 infants born to asthmatics and *n* = 371 born to non‐asthmatics. Figure 2 shows the network analysis between separate proteins in both groups. Protein networks in cord blood from infants born to asthmatics showed a higher number of edge connections and stronger relationships (higher Spearman's correlation coefficients) than networks from infants born to non‐asthmatics (correlation coefficients are shown in Figure S3). Even though the connections were similar, the centrality parameters were different between the groups. Infants born to asthmatics had the highest node centrality with PDGF‐AA, TGF‐β1, IFN‐γ, MMP‐9, and p62 at different levels, while infants born to non‐asthmatics had many linked proteins that were part of the most central, including EGF, IFN‐γ, IL‐13, IL‐8, and PDGF‐AA (Figure 3). Although all these infants are asymptomatic at birth, the connectivity structures of the correlation networks were different in strength.

When taking the results of both approaches together—the direct comparison of protein levels using Tobit regression, and the correlation network—we prioritized IFN‐γ as the most discriminative protein marker representing the functional category “inflammation,” p62 for “autophagy/oxidative stress,” and MMP‐9 for “ECM‐turnover.”

### 3.1 Infant Lung Function at 4–6 Weeks of Age (Second Aim, Part One)

Out of the 715 term infants with cord blood markers available, 495 infants underwent tidal or MBW measurements at 4–6 weeks of age that were technically acceptable. This subset differed from the overall cohort only in gestational age and mode of delivery; differences in birth weight and length persisted. In addition, at the time of the test, infants born to asthmatics were significantly older regarding postmenstrual age (45.9 weeks vs. 44.9 weeks, *p* < 0.001), and therefore heavier (4.7 kg vs. 4.4 kg, *p* < 0.001) and longer (55.8 cm vs. 54.6 cm, *p* < 0.001), and fewer were breastfed (74.7% vs. 97.1%, *p* < 0.001, Table S5).

**FIGURE 4:**
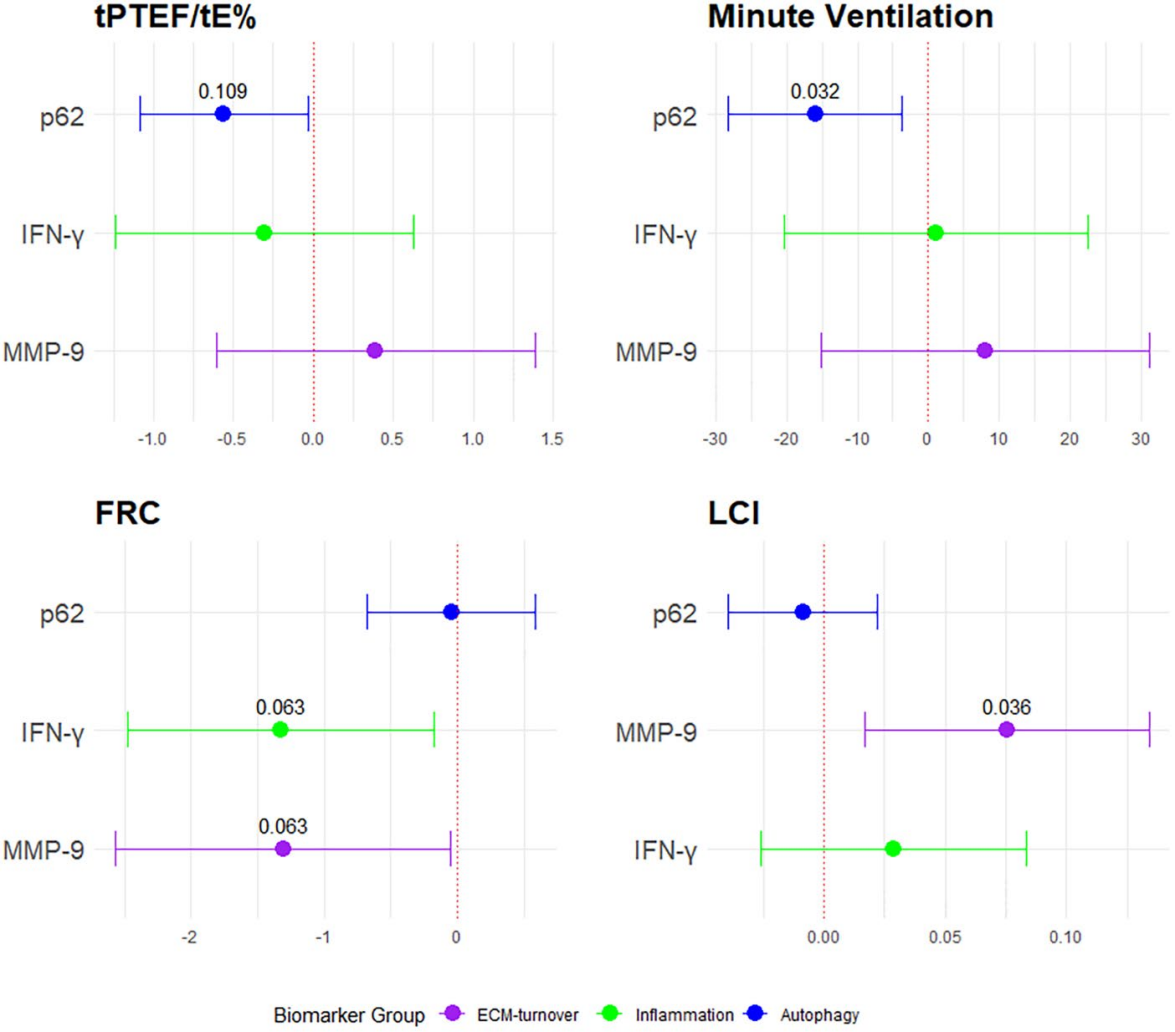
Coefficient estimates from multivariable linear regression models showing the associations between three representative markers as exposures and lung function parameters measured at 4–6 weeks. ECM‐turnover, extracellular matrix turnover; FRC, functional residual capacity; IFN‐γ, interferon gamma; LCI, lung clearance index; MMP‐9, matrix metalloproteinase 9; p62, ubiquitin‐binding protein sequestosome 1; tPTEF/tE%, the ratio of time to peak tidal expiratory flow as a percentage of total expiratory time. Estimates are reported as a coefficient with corresponding 95% CI confidence interval according to protein levels as exposure and adjusted for maternal asthma during pregnancy, sex, having siblings at birth, postmenstrual age at lung function test in weeks, weight at test, maternal smoking during pregnancy, mode of delivery, study centre, and time of cord blood processing in days. *p*‐values shown are after Benjamini–Hochberg multiple comparison correction.

We found an inverse association between p62 levels and minute ventilation (*β* −16.18, 95% CI, −28.44 to −3.91, *p*_adj_ = 0.032). We also found a positive association between MMP‐9 levels and LCI (*β* 0.07, 95% CI, 0.02–0.14, *p*_adj_ = 0.036). FRC values were inversely associated with IFN‐γ (*β* −1.26, 95% CI, −2.41 to −0.11) and MMP‐9 levels (*β* −1.27, 95% CI, −2.53 to −0.01, Figure 4). tPTEF/tE% values were inversely associated with levels of p62 (*β* −1.37, 95% CI, −2.72 to −0.04). However, after applying Benjamini–Hochberg adjustment, these associations became marginally non‐significant: FRC and IFN‐γ *p*_adj_ = 0.063, FRC and MMP‐9 *p*_adj_ = 0.063, tPTEF/tE% and p62 *p*_adj_ = 0.108 (Table S6).

### 3.2 Bronchiolitis Hospitalization (Second Aim, Part Two)

Out of the 715 term infants with cord blood markers available, *n* = 605 had information on bronchiolitis hospitalization (*n* = 22 cases and *n* = 583 non‐cases). Those infants only differed by sex (72.7% male vs. 49.7% female, *p* = 0.049) and birth order (only 22.7% of cases were first born compared to 47.3% of the non‐cases, Table S7).

To investigate the association of IFN‐γ, p62, and MMP‐9 with the incidence of bronchiolitis hospitalization, we performed logistic regressions adjusting for confounders. We found a protective effect of higher MMP‐9 on bronchiolitis risk (aOR 0.45, 95% CI, 0.27–0.73, *p*_adj_ = 0.001, Table 2). No associations were found for IFN‐γ or for p62.

**TABLE 2:** Association between cord blood proteins and bronchiolitis hospitalization in the first year of life while adjusting for being born to an asthmatic mother and other confounders.

	Bronchiolitis hospitalization (*n* = 22 cases, *n* = 583 non‐cases)			
	aOR	95% CI	*p*	*p*_adj_
IFN‐γ	0.85	0.61–1.18	0.333	0.363
P62	1.16	0.84–1.61	0.150	0.281
MMP‐9	**0.47**	**0.29–0.77**	**0.002**	**0.004**

*Note:* Logistic regression models were used and adjusted for asthma in pregnancy, sex, birth order, having siblings at birth, gestational age in weeks, birth weight in *Z*‐score, season of birth in cosine function, maternal smoking during pregnancy, mode of delivery, study center, and time of cord blood processing in days. *p*‐values < 0.05 are shown in bold. Adjusted *p*‐values were calculated by Benjamini–Hochberg method.

Abbreviations: 95% CI, confidence interval; Adj., adjusted; aOR, adjusted odds ratio.

### 3.3 Asthma in Childhood (Second Aim, Part Three)

Out of the 715 infants with markers available, *n* = 421 were followed up at six years of age, with *n* = 29 parent‐reported asthma diagnosis cases (*n* = 392 non‐cases). The asthmatic group was more commonly born to asthmatics (55.2% vs. 8.9%, *p* < 0.001), and they were heavier (3.6 kg vs. 3.4 kg, *p* = 0.020) and longer (56.1 cm vs. 54.6 cm, *p* = 0.002) at birth (Table S8). Using logistic regression adjusting for confounders, we also found a protective effect of higher MMP‐9 on asthma in childhood risk (aOR 0.53, 95% CI, 0.32–0.86, *p*_adj_ = 0.033, Table 3). No associations were found for IFN‐γ nor for p62.

**TABLE 3:** Association between cord blood proteins and doctor‐diagnosed asthma in childhood while adjusting for being born to an asthmatic mother and other confounders.

	Asthma in childhood (*n* = 29 cases, *n* = 392 non‐cases)			
	aOR	95% CI	*p*	*p*_adj_
IFN‐γ	0.73	0.47–1.14	0.165	0.247
P62	1.00	0.76–1.31	0.991	0.991
MMP‐9	**0.53**	**0.33–0.86**	**0.011**	**0.033**

*Note:* Logistic regression models were used and adjusted for asthma in pregnancy, sex, birth order, having siblings at birth, gestational age in weeks, birth weight in *Z*‐score, season of birth in cosine function, maternal smoking during pregnancy, mode of delivery, study center, and time of cord blood processing in days. *p*‐values < 0.05 are shown in bold. Adjusted *p*‐values were calculated by Benjamini–Hochberg method.

Abbreviations: 95% CI, confidence interval; Adj., adjusted; aOR, adjusted odds ratio.

To further confirm our results, we calculated the variance inflation factors (VIFs) for all covariates in our regression models. The VIFs for maternal asthma and study site were 3.49 and 3.53, respectively. Other covariates showed minimal collinearity with VIFs close to 1. Sensitivity analysis included models excluding infants born by cesarean section (Table S9) and similar results were found for those models. Additionally, we performed LASSO regression, adjusting for variables identified as relevant by the method, and found similar results. IL‐17A and VEGF‐AA associations remained significant after Benjamini–Hochberg adjustments (Table S13).

## 4 Discussion

Although these infants are asymptomatic at birth, for the first time, we identified significant differences in cord blood protein fingerprints between asymptomatic term infants born to mothers with asthma during pregnancy and asymptomatic term infants born to non‐asthmatic mothers. These markers might be related to lung development in utero including airway formation, branching, and alveolar development, and to autophagy/oxidative stress response (p62) [38], inflammation (IFN‐γ) [39], and ECM‐turnover (MMP‐9) [14]. Correlation network analysis revealed strong interdependencies suggesting a complex interplay. We thus show that IFN‐γ, p62, and MMP‐9 were associated with lung function early in life. In addition, we found that lower MMP‐9 was associated with the risk of bronchiolitis hospitalization in the first year of life and asthma in childhood. This new evidence supports the hypothesis that the in utero environment of offspring from asthmatics impacts postnatal respiratory health. Our results add to recent findings that impaired early‐life lung function is a risk factor for later chronic respiratory morbidity [40, 41, 42, 43, 44].

Studies on fetal growth—including fetal size, weight, and prematurity—have identified key periods relevant for asthma and other respiratory disease development [45]. Lung development studies highlight an intricate involvement of signaling pathways [46] and interactions of multiple cytokine‐secreting immune cells [47]. For instance, some evidence from human models indicates that inflammatory cytokines, such as IFN‐γ, play a role in normal term pregnancy and fetal growth and development. The Th1/Th2 paradigm in adult allergic disease seems not to be applied in infants, where a stronger Th1 and Th2 bias has been demonstrated, with deficient production of IFN‐γ, IL‐4, and IL‐17A [48]. Reduced levels of IFN‐γ may indicate immature innate and adaptive immune responses to inflammatory stimuli or infection—in earlier studies the same mechanisms responsible for impaired cytokine production in genetically at‐risk infants are already active during late fetal development [49]. Previously, we have shown that, in babies born to asthmatics, activated type 2 innate lymphoid cells in the cord blood were associated with reduced tPTEF/tE% and increased LCI at six weeks of age [8]. Our observations in the current study similarly show distinct protein levels and interactions in the cord blood of offspring of asthmatics.

We also found higher levels of p62, and lower levels of Beclin‐1, which may reflect altered autophagy in infants born to asthmatics. Basal‐level autophagy is crucial for cellular homeostasis and lung development, including branching morphogenesis and alveolar formation [50]. It regulates mesenchymal cell proliferation and differentiation and contributes to surfactant homeostasis. When dysregulated, autophagy can cause hypoxia, inflammation, and oxidative stress [51]. p62 deletion following prolonged hyperoxia leads epithelial cells into a stage of unsalvageable cell death [52]. Autophagy dysregulation has been linked to pregnancy complications, including preeclampsia [53] and prematurity [35]. For instance, our latest work in the BILD study comparing term and preterm infants also found higher levels of p62. Despite these interesting findings in another risk group, due to the nature of our human samples, we cannot conclude the functional significance of autophagic markers measured in cord blood plasma after birth. Further research is needed to clarify these results in plasma, including samples from other biological fluids [54].

Metalloproteinases (MMPs) are a major group of enzymes that regulate the composition of the extracellular matrix and are involved in lung branching morphogenesis, homeostasis, repair, tissue remodeling, inflammation, and wound healing [14, 55], operating in a stimulus‐specific manner either causing damage or repair [56, 57]. Among them, MMP‐9 is capable of degrading components of the cellular matrix and contributes to tissue remodeling in adult respiratory disease, and its production varies depending on asthma phenotype [58, 59]. However, it remains unclear whether MMP‐9 is causal in lung remodeling or part of the inflammatory and reparative response. Our results show lower levels of MMP‐9 in infants born to asthmatic mothers, and we speculate that varying levels of MMP‐9 during development could have vastly different consequences later in life [60]. There is a significant lack of data regarding MMP‐9 expression in normal human fetal lungs; moreover, most existing studies focus on pathological conditions such as bronchopulmonary dysplasia [61] and pulmonary hypoplasia [62]. However, our study corroborates findings from Kraljevic et al. in fetal human lungs, suggesting that MMP‐9 expression in the underlying mesenchyme of developing alveoli was likely essential for proper vascular development and the formation of sufficient surface area for effective oxygen exchange [63]. Our observation does not allow inferences of involved mechanisms; nevertheless, IFN‐γ and MMP‐9 levels showed the largest differences when stratified by infants born to asthmatics and non‐asthmatics during pregnancy.

We speculate that the differences found in our analysis may be linked to impaired lung development in offspring. Minute ventilation was inversely associated with p62, and LCI was positively associated with higher MMP‐9. We also observed an association between lower tPTEF/tE% and higher p62, which was only marginally significant after adjusting for multiple comparisons. Nevertheless, these findings support our hypothesis that impaired autophagy may be associated with airway obstruction.

Our previous findings indicated lower tPTEF/tE% in male infants born to asthmatic mothers, and our current results confirm this finding even in non‐stratified analysis [3]. Interestingly, we now observe a marginally significant decrease in lung volume (FRC) with each unit increase in MMP‐9 and IFN‐γ. Given the highly dynamic nature of end‐expiratory lung volumes in infants, we speculate that lower FRC may reflect lower pulmonary compliance and adaptive breathing (trend to lower tPTEF/tE%) with shortened expiratory time as a mechanism to avoid lung collapse [64]. Further studies with a larger infant lung function dataset or histological analysis are warranted to explore these mechanisms in more detail.

In this current study, we found an association between higher MMP‐9 levels at birth and lower risk of bronchiolitis in the first year of life, as well as reduced asthma incidence at six years. Bronchiolitis is the leading cause of lower respiratory tract infection resulting in hospitalization in infants, with an incidence ranging from 18% to 32% [65]. In our previous analysis of the BLT cohort, we found that reduced infant lung function was associated with infant bronchiolitis [33] and that higher eosinophil and lower neutrophil levels in cord blood were associated with increased bronchiolitis risk in babies born to mothers with asthma during pregnancy [9]. While our observational data do not allow for causal inference, we hypothesize that MMP‐9 may be necessary for better lung organogenesis and angiogenesis, leading to better airway branching [62]. Theoretically, altered lung growth could mediate susceptibility to airway infection; however, our sample size limits reliable statistical mediation analysis.

Notably, MMP‐9 genes have been implicated in granulocyte responses during lower respiratory tract infection [66] and respiratory syncytial virus (RSV) infectivity [67]. Additionally, adults with asthma show deficient MMP‐9 release in response to bacterial proteins that stimulate neutrophil migration compared to healthy controls. Further research is warranted to clarify these mechanisms. We found no association between IFN‐γ or p62 and bronchiolitis hospitalization or asthma in childhood, which contrasts with findings from Mondell et al. [68], who show a link between IFN‐γ and lower respiratory tract infection. These differences may be because our study focuses solely on bronchiolitis cases, whereas theirs included bronchiolitis, bronchitis, or pneumonia cases in infants aged 0–12 months.

Strengths of our analysis include the aligned methodologies between the BILD and BLT studies such as cord blood analysis [35], lung function technology [3], and their standards [26]. By combining these datasets, we gained the unique opportunity of investigating the in utero differences between mothers with asthma during pregnancy versus non‐asthmatics, as evidenced by cord blood protein profiles. In addition, we only included term infants, precluding bias, since asthma is associated with prematurity which may be related to MMP‐9 [69]. Our findings were further strengthened by the ability to examine the relationship between these markers and clinical outcomes. Additionally, to avoid statistical multiple comparison issues, we reduced the number of proteins (exposure) and adjusted for multiple comparison effects: future studies should explore broader immune profiles. Limitations include that the role of some of the protein markers (autophagy‐related) is better understood in cell and tissue cultures, with only sparse evidence recently describing their role in plasma [70]. Additionally, we measured the proteins only at birth, but many confounders may be linked with later lung function and bronchiolitis. Therefore, studies investigating the markers at the time of the lung function tests could provide further insight into the associations we found. Although this is likely one of the largest datasets with these parameters, future studies could benefit from even larger samples of infants born to asthmatics.

Our study is the first to identify proteins at birth that are different in asymptomatic term infants born to mothers with asthma during pregnancy compared to those born to non‐asthmatics. This supports the hypothesis that an impaired intrauterine environment in asthmatic mothers involving autophagy/oxidative stress‐, inflammatory‐, and ECM‐turnover‐related proteins may contribute to impaired postnatal lung function and respiratory morbidity in the first year of life and asthma in childhood. These findings contribute to the hypothetical concept that “intrauterine programming” may contribute to the evolution of chronic respiratory disease in the offspring.

## Author Contributions

Conception and design: C.R.D.S.S., U.F., A.C., J.M. Data collection and analysis: C.R.D.S.S., G.M.C.G., N.K., O.G., A.M., F.W., P.D.R. Data interpretation: C.R.D.S.S., U.F. Manuscript drafting: C.R.D.S.S., U.F. Manuscript revision and final approval: C.R.D.S.S., G.M.C.G., N.K., O.G., A.M., S.S., F.W., V.E.M., P.D.R., P.D.S., J.U., B.S., R.S., S.Y., L.M., P.L., P.S., P.G.G., J.M., A.C., U.F.

## Ethics Statement

In both cohorts, written informed parental consent was obtained at the time of enrolment. Both studies are approved by the local Human Ethics Committees.

## Conflicts of Interest

The authors declare no conflicts of interest.

## Supporting information


**Appendix S1:** all70044‐sup‐0001‐AppendixS1.docx.

## Data Availability

The data that support the findings of this study are available from the corresponding author upon reasonable request.
