# Supplementary material for: Markers in Infants of Mothers With Asthma and Associations With Respiratory Outcomes
Source: Allergy. 2025 Sep 15;81(1):145–56. doi: 10.1111/all.70044 (PMC12773685; doi:10.1111/all.70044)
Supplement: Supplementary file 1 — Appendix S1: all70044‐sup‐0001‐AppendixS1.docx. [file ALL-81-145-s001.docx]

**SUPPLEMENTAL MATERIAL**

**Markers in infants of mothers with asthma and associations with respiratory outcomes** Carla Rebeca Da Silva Sena^1,2,3^, Gabriela Martins Costa Gomes^2^, Noëmi Künstle^1^, Olga Gorlanova^1^, Andrea Marten^1^, Sven Schulzke^1^, Florian Wyler^3^, Vanessa E Murphy^2^, Paul D Robinson^4,5^, Peter D Sly^5^, Jakob Usemann^1^, Benjamin Stoecklin^1^, Ruth Steinberg^3^, Sophie Yammine^3^, Loretta Müller^3,9^, Philipp Latzin^3^, Pablo Sinues^1^, Peter G Gibson^2,6,8^, Joerg Mattes^2,7^, Adam Collison^2^, Urs Frey^1^

*^1^ University Children's Hospital Basel UKBB, University of Basel, Basel, Switzerland*

*^2^* *Asthma & Breathing research program, Hunter Medical Research Institute, Newcastle, University of Newcastle, New South Wales, Australia*

*^3^ Division of Paediatric Respiratory Medicine and Allergology, Department of Paediatrics, Inselspital, Bern University Hospital, University of Bern, Bern, Switzerland*

*^4^ Department of Respiratory Medicine, Queensland Children’s Hospital, South Brisbane, Queensland, Australia*

*^5^ Children’s Health and Environment Program, Child Health Research Centre, The University of Queensland, South Brisbane, Queensland, Australia*

*^6^ Respiratory & Sleep Medicine Department, John Hunter Hospital, Newcastle, New South Wales, Australia*

*^7^ Paediatric Respiratory & Sleep Medicine Department, John Hunter Children’s Hospital, Newcastle, New South Wales, Australia*

*^8^* *School of Medicine and Public Health, College of Health, Medicine and Wellbeing, University of Newcastle, New South Wales, Australia*

*^9^ Lung Precision Medicine (LPM), Department for BioMedical Research (DBMR), University of Bern, Bern, Switzerland*

**#Correspondence:**

Prof Urs Frey

E-mail: [urs.frey@ukbb.ch](mailto:urs.frey@ukbb.ch); Address: University Children’s Hospital Basel, Spitalstrasse 33, 4056 Basel, Switzerland

# **METHODS**

*Population Characteristics*

The Breathing for Life Trial (BLT) birth cohort (https://www.breathingforlife.com.au) includes offspring of mothers with mild to moderate asthma during pregnancy who were randomized to two different asthma management groups in pregnancy (1). Pregnant women were recruited from six Australian public hospital antenatal clinics (7 March 2013 to 11 June 2019). Participants had doctor-diagnosed asthma, symptoms of asthma, or asthma medication use, or both (prior 12 months), were aged ≥18 years and were between 12 and 23 completed weeks of gestation at randomization. Regarding the primary BLT outcome in the pregnancy RCT, there was no difference in the effect of asthma management guided by exhaled nitric oxide levels, versus usual care, on perinatal outcomes (23). For the BLT study infants were born between May 2014 and December 2019.

The prospective Basel-Bern Infant Lung Development (BILD) cohort (https://www.bild-cohort.ch) comprises unselected neonates recruited since 1999 in the region of Bern and since 2012 in Basel, Switzerland (23, 24). For this study we included infants born between April 1999 and July 2023. Recruitment of infants happened prenatally or shortly after birth resulting in n=802 infants who had cord blood collected at birth. Additional exclusion criteria for the current study were differences in sampling procedure for cord blood collection, such as missing clinical or sociodemographic data, time from blood collection to processing (e.g., centrifugation) >3 days or insufficiently documented sampling procedure. After exclusion, the sample consisted of n=686 term infants. Infants were followed up at 4–6 weeks of corrected age and underwent lung function testing. Additional to the follow-up visit, infants were followed up weekly through phone calls. A history of maternal asthma was defined as self-reported, doctor-diagnosed asthma in the questionnaire or during the interview. Maternal asthma during pregnancy was of mild to moderate severity. Potential risk factors were assessed through interviews using standardized questionnaires.

*Cord blood proteins (first aim)*

Exposures were selected cord blood protein marker fingerprints involved in lung development as well as in-utero oxidative stress response, inflammation, and extracellular matrix turnover (ECM-turnover). Immediately after birth, umbilical cord blood was collected into ethylenediaminetetraacetic acid (EDTA) tubes for plasma. Samples were centrifuged and plasma was stored at −80 °C until further analysis. BLT samples were processed within six hours of the birth, and BILD samples within 72 hours. Samples were excluded from this analysis if processing time was more than 72 hours. Plasma proteins were analyzed with Enzyme-Linked Immunosorbent Assay (ELISA) or Luminex. Samples from Australia were shipped to Switzerland and analyses were performed in one center. All samples were thawed and aliquoted into 96-well-plates and thawed again before the assay. This limited freeze–thaw cycles to a minimum, since on the day of running the assays, only the plate that was needed for the specific assay of that day had to be freshly thawed. Autophagy markers were measured in two distinct phases: the BILD cohort in 2019 and the combined BILD + BLT cohorts in 2023. To adjust for potential batch effects due to this timing difference, we applied formal batch correction using ComBat (R package sva) prior to analysis. For all other proteins, samples were processed and analyzed simultaneously under uniform laboratory conditions, so no batch correction was necessary.

For all marker analyses, the manufacturer’s protocols were strictly followed, and detection limits reported by the manufacturer were used. Due to differences in the sample volume, the markers have varying sample sizes. Protein concentrations along with limit of detection (LOD) in the cord blood are given in Table S3.

*Analysis of inflammation and ECM-turnover markers by Luminex*

Inflammation markers (IL-8, IL-1β, TNF-α, IFN-γ) and remodeling (PDGF-AA, IL-4, IL-13, IL-17, and TGF-β1) were measured using a protein level by Luminex® xMAP® Technology for Multiplexed Single-Well Analyses using the MILLIPLEX® Human Cytokine/Chemokine/Growth Factor Panel A Magnetic Bead Panel (HCYTA60K) (Merck-Millipore, Burlington, MA). Multiplex-based assay read-out was performed using INTELLIFLEX® system with the Instrument SW Version: 2.0.1017 (Merck-Millipore, Burlington, MA) in accordance with the manufacturer’s instruction with 2 hours incubation of the plasma samples with antibody-immobilized premixed magnetic beads. Final analysis was carried out with the Belysa™ Immunoassay Curve Fitting Software V1 (Merck-Millipore, Burlington, MA) (2).

*Analysis of ECM-turnover and autophagy markers by ELISA.* We analyzed the ECM-turnover matrix metalloproteinase 9 (MMP-9) using Human MMP-9 ELISA kits (FineTest Biotech Inc., Wuhan, China, EH0238) (3). All assays employed a high-affinity [Biotin](https://www.sciencedirect.com/topics/medicine-and-dentistry/biotin) conjugated specific antibody and [Streptavidin](https://www.sciencedirect.com/topics/medicine-and-dentistry/streptavidin) Biotin complex as part of the detection system to generate and measure optical density (OD). The OD absorbance at 450 nm was read using the BioTek Synergy H4 Hybrid Reader and the Gen5 2.00 analysis software to determine the concentration using the standards.

Autophagy markers included Beclin-1 (Beclin-1), ubiquitin-binding protein sequestosome 1 (p62/SQSTM1, referred to hereafter as p62), sirtuin 1 (SIRT1) measurements were done in 2019 and 2024 (4-6). The analysis was performed in plasma with ELISA Kits (AVIVA Systems Biology, San Diego, CA) for Beclin-1 and SIRT1, and ELISA Kits (ENZO Life Sciences, Farmingdale, NY) for p62 (7). Autophagy protein 5 (ATG5) was analyzed using the Human ATG5 ELISA Kit (FineTest Biotech Inc., Wuhan, China, EH1729).

*Infant lung function at 4–6 weeks of age (second aim, part one).* Infant lung function was performed supine during behaviorally defined quiet natural sleep according to the European Respiratory Society/American Thoracic Society (ERS/ATS) standards of infant lung function testing (8, 9), using an infant mask (sizes 0, 0/1, and 1; Homedica, Huenenberg, Switzerland). Mask-size dead space was corrected during analysis. Flow was measured using an ultrasonic flow meter in both cohorts (Spiroson; Eco Medics, Duernten, Switzerland). Testing protocols between sites only differed in the duration of testing. In the BLT study, tidal breathing flow volume loop (TBFVL) was performed for 90 seconds to obtain at least 30 good-quality breaths (4, 29), while in the BILD cohort TBFVL was performed with the purpose of obtaining 100 good-quality breaths (10). For the BLT cohort, data were included if no apparent volume drift (defined as a change of <3 mL/s over at least 30 breaths) was present (9).

*Bronchiolitis hospitalization (second aim, part two).* For the BLT cohort, bronchiolitis hospitalizations during the first year of life were identified in the electronic medical records held for each participant within the local health district networks of New South Wales (NSW) Health, Australia. Infants with missing information were excluded. In the BILD study, respiratory symptoms (including wheezing and lower respiratory tract symptoms) were assessed using a standardized score that groups symptoms into four levels according to severity, during weekly phone calls in the first year of life (11). Then extensive verification of weekly phone call notes was performed to identify infants with a history of bronchiolitis. Subsequently, medical records were checked to ascertain infants who were admitted or presented to the emergency department (ED) with a clinical diagnosis of bronchiolitis.

*Asthma in childhood (second aim, part three).* Both cohorts conducted follow-up assessments at six years of age. Parents were interviewed to determine whether a doctor had diagnosed their child with asthma. Additionally, standardized International Study of Asthma and Allergies in Childhood (ISAAC) questionnaires were used to assess respiratory symptoms and medication use in the past 12 months (references 12–14). For the BLT cohort, parents were asked: “Has your child been diagnosed with asthma by a doctor?” with a follow-up question regarding the age at diagnosis. In the BILD cohort, the question posed was: “Have you ever been told by a doctor that your child has asthma?”. The asthma diagnoses reported by parents were subsequently verified by a pediatric pulmonologist, who reviewed the clinical information provided during the interview.

**Statistical analysis**

To account for potential batch effects in our data, as some of the autophagy markers (p62, Beclin-1, SIRT1) were measured in 2019 and some in 2024, we employed the ComBat algorithm to correct batch-related variations caused by differences in experimental conditions rather than true biological differences. For those three markers (p62, SIRT1 and Beclin-1) we used the ComBat corrected values log2-transformed (12).

*Group comparison of cord blood protein markers using Tobit regression analysis (first aim, part one).* Protein levels were compared using Tobit regression where maternal asthma during pregnancy was treated as the primary exposure in the model, rather than grouping by classification, due to sample size limitations. Models were further adjusted for sex, having siblings at birth, gestational age, maternal smoking during pregnancy, mode of delivery (vaginal or Cesarean section), birth weight (z-score), study center, and time to processing sample in days.

*Direct group comparison of cord blood protein markers using correlation network analysis (first aim, part two*). To identify which threshold to use for the network analysis we calculated the change in the centrality measures by a function of R2 and chose the threshold based on the flattening of the curve (R2 ≥0.3). To compare the networks, we calculated the difference between the measures of centrality between the groups.

*Association of proteins with lung function (second aim, part one).* Using linear regression analysis, we tested the association between the protein levels (exposure) and postnatal lung function measurements (outcome), adjusting for maternal asthma during pregnancy, sex, having siblings at birth, postmenstrual age at lung function, maternal smoking during pregnancy, mode of delivery, weight at visit (in grams), breastfeeding up to visit day, study center, and time to sample processing (in days), based on our previous studies (3, 37).

*Bronchiolitis hospitalization (second aim, part two).* Bronchiolitis hospitalization risk was calculated using logistic regression, with protein levels as the exposure. Results are expressed as adjusted odds ratio (aOR) with 95% confidence intervals (CI) (38). Models were adjusted for maternal asthma during pregnancy, sex, birth order, gestational age at birth, maternal smoking during pregnancy, mode of delivery, season of birth, birth weight (z-score), and study center.

*Asthma in childhood (second aim, part three).* Asthma risk was calculated using logistic regression, with protein levels as the exposure. Results are expressed as adjusted odds ratio (aOR) with 95% confidence intervals (CI) (38). Models were adjusted for maternal asthma during pregnancy, sex, birth order, gestational age at birth, maternal smoking during pregnancy, mode of delivery, season of birth, birth weight (z-score), and study center.

*Sensitivity analysis.* To test for the robustness of our models, we conducted the analysis only including children born from vaginal birth, as Cesarean section mode of delivery was a significant confounder associated with the main biomarkers (Table S8). Additionally, we performed LASSO regression, adjusting for variables identified as relevant by the method, and found similar results. For the asthma model, we also included in the model any history of hospitalization for bronchiolitis.

Supplementary Tables

Table S1: In-utero function of proteins measured.

| Protein | In-utero biological function |
| --- | --- |
| *Inflammatory associated* | |
|  |  |
| IL-1β | - Mediator of the inflammatory response, including cell proliferation, differentiation, and apoptosis. - Decreases SOX9 expression and proliferation, resulting in airway differentiation. (13) |
| IL-8 | - Proinflammatory cytokine, with specific chemotactic activity that brings neutrophils to inflammatory site. - May promote production of fetal lung surfactant when in high concentration in mice lung. (14) |
| TNF-α | - Induction of cell proliferation, differentiation and apoptosis. - Regulates several biological processes in organogenesis including airway development acting on airway epithelial cells. - Promotes lung branching morphogenesis and expression surfactant proteins in embryonic mice. (15) |
| IFN-γ | - Proinflammatory cytokine. - Mediates uterine spiral artery remodeling. - Immune response of the lung to infection. - Plays critical roles that include initiation of endometrial vasculature remodeling, angiogenesis at implantation sites, and maintenance of the decidual (maternal) component of the placenta. (16) |
| IL-4 | - Enhances remodeling. - Essential for branching morphogenesis. - Induces type-2 mediated macrophage activation. (17) |
| IL-13 | - Involved in angiogenesis, promoting optimal alveolarization - Essential for branching morphogenesis. (17) |
| IL-17 | - Proinflammatory. - Promotes survival, proliferation, and invasion of human trophoblast cells during the first trimester of pregnancy. (17) |
| *Autophagy/ Oxidative stress proteins* | |
| p62 | - Accumulation can indicate inefficient autophagy. (18) |
| Beclin-1 | - Intrinsic autophagy is critical for proper lung development and morphogenesis. - Abrogation of intrinsic autophagy in vitro, in vivo, or both, during either early or late gestation, disrupts airway branching as well as sacculi formation and delays maturation of the lung - Autophagosome formation (19) |
| SIRT1 | - Overexpression of SIRT1: reverses senescence, negatively regulates the expression of several senescence-associated secretory phenotype (SASP) factors. - Includes IL-8 and IL-1β among the regulated SASP factors |
| ATG5 | - Required for the formation of the autophagosome and the subsequent induction of autophagy. |
| *ECM-turnover/modelling associated* | |
| MMP-9 | - Critical agent facilitating extracellular matrix remodeling: - Destabilizes the matrix, - Activates collagen decomposition processes. - MMPs play critical roles in lung organogenesis (20) |
| PDGF-AA | - Is part of the epithelial-mesenchymal signaling in the developing lung, including branch initiation (21) |
| TGF-β | - Enhances remodeling. - Essential for branching morphogenesis. - Guide lung development in part by inducing the expression of matrix-degrading metalloproteinases. (22) |
| EGF | - Essential for branching morphogenesis. - Guide lung development in part by inducing the expression of matrix-degrading metalloproteinases (22) |
| VEGF-A | - Implicated in vascularization. - Essential for branching morphogenesis (22) |

*Abbreviations*: IL-1β—interleukin 1β, IL-8—interleukin 8, TNFα—tumor necrosis factor α, IFN-γ—interferon gamma, p62—protein Sequestosome 1, Beclin-1—Beclin-1, SIRT1—Sirtuin 1, ATG5—Autophagy protein 5, ECM-turnover— extracellular matrix turnover, MMP-9—matrix metalloproteinase-9, PDGF-AA—platelet-derived growth factor AA, TGF-β1—transforming growth factor β1, IL-4—interleukin 4, IL-13—interleukin 13, IL-17A—interleukin 17A, EGF—epidermal growth factor, VEGF-A—vascular endothelial growth factor A.

Table S2: Demographic data of all infants and comparison between infants in the BILD versus BLT cohort.

|  | **Total**  **(n=715)** | **BILD**  **(n=612)** | **BLT**  **(n=103)** | **p-value** |
| --- | --- | --- | --- | --- |
| **Maternal baseline characteristics** |  |  |  |  |
| Maternal smoking during pregnancy *n (%)* | 54 (7.5%) | 40 (6.5%) | 14 (13.6%) | **0.024** |
| Asthma during pregnancy *n (%)* | 135 (18.8%) | 32 (5.0%) | 103 (100%) | **<0.001** |
| **Infant baseline characteristics** |  |  |  |  |
| First born *n (%)* | 333 (46.6%) | 280 (45.8%) | 53 (52.0%) | 0.284 |
| Male *n (%)* | 366 (51.2%) | 311 (50.8%) | 55 (53.4%) | 0.671 |
| Delivery type: |  |  |  | **0.027** |
| Vaginal *n (%)* | 553 (77.3%) | 483 (78.9%) | 70 (69.3%) |  |
| Cesarean section *n (%)* | 156 (21.8%) | 136 (20.6%) | 31 (30.7%) |  |
| Season of birth: |  |  |  | **0.025** |
| Winter *n (%)* | 125 (17.5%) | 96 (15.7%) | 29 (28.2%) |  |
| Spring *n (%)* | 220 (30.8%) | 189 (30.9%) | 31 (30.1%) |  |
| Summer *n (%)* | 170 (23.8%) | 150 (24.5%) | 20 (19.4%) |  |
| Fall *n (%)* | 200 (28.0%) | 177 (28.9%) | 23 (22.3%) |  |
| Gestational age in weeks † | 39.7 (1.13) | 39.8 (1.11) | 39.3 (1.78) | **<0.001** |
| Weight at birth, kg | 3.40 (0.5) | 3.38 (0.44) | 3.51 (0.50) | **0.005** |
| Weight at birth, z-score | -0.14 (0.89) | -0.20 (0.86) | 0.24 (0.96) | **<0.001** |
| Length at birth, cm | 49.9 (2.28) | 49.7 (2.07) | 51.6 (2.82) | **<0.001** |
| Lung function |  | n=420 | n=75 |  |
| Postmenstrual age at test | 45.0 (1.3) | 44.9 (1.2) | 46.5 (1.6) | **<0.001** |
| Length at test, cm | 54.9 (2.5) | 54.7 (2.4) | 56.6 (2.6) | **<0.001** |
| Weight at test, kg | 4.5 (0.6) | 4.4 (0.6) | 5.0 (0.7) | **<0.001** |
| tPTEF/tE% | 36.0 (10.7) | 36.3 (10.7) | 31.5 (10.6) | **0.002** |
| Minute ventilation | 1429.7 (267.2) | 1417.9 (259.0) | 1546.4 (318.7) | **0.001** |
| FRC | 87.3 (0.6) | 87.2 (12.2) | 89.4 (14.9) | 0.359 |
| LCI | 6.7 (0.6) | 6.7 (0.6) | 6.9 (0.6) | **0.022** |
| Bronchiolitis hospitalization *n (%)* | 28 (4.1%) | 24 (4.0%) | 4 (4.0) | 0.954 |

Categoric variables are presented as counts and percentages; continuous variables are presented as means with SDs and † and as median with IQR. Groups were compared using either a t-test or a chi-square, as appropriate. P-values <0.05 are shown in bold. Abbreviations: BILD—Basel-Bern Infant Lung Development; BLT—Breathing for Life Trial; tPTEF/tE% the ratio of time to peak tidal expiratory flow as a percentage of total expiratory time, FRC functional residual capacity, LCI lung clearance index.

Table S3: Distribution of proteins in the cord blood of infants and their level of detection (LOD)

| **Proteins** | N | Mean (SD)  Raw data | Median (IQR)  Raw data | LOD | Detection %  >LOD | | Mean (SD)  Log2 | |  |  |  |  |
| --- | --- | --- | --- | --- | --- | --- | --- | --- | --- | --- | --- | --- |
| **Inflammatory associated** |  |  |  |  | | | |  |  |  |  |  |
| IL-1β, pg/mL | 597 | 14.92 (127.57) | 4.91 (10.34) | 1.1 | 72.53 | | 1.93 (2.08) | |  |  |  |  |
| IL-8, pg/mL | 597 | 42.58 (295) | 5.08 (10.91) | 0.24 | 99.66 | | 2.60 (2.28) | |  |  |  |  |
| TNF-α, pg/mL | 597 | 56.6 (24.02) | 52.94 (25.07) | 4.4 | 99.5 | | 5.69 (0.68) | |  |  |  |  |
| IFN-γ, pg/mL | 597 | 37.69 (26.92) | 31.23 (35.58) | 1.29 | 98.49 | | 4.78 (1.32) | |  |  |  |  |
| IL-4, pg/mL | 597 | 3.22 (3.55) | 2.06 (3.26) | 0.25 | 92.29 | | 0.86 (1.73) | |  |  |  |  |
| IL-13, pg/mL | 597 | 30.3 (30.65) | 21.7 (38.5) | 2.13 | 83.75 | | 3.90 (2.08) | |  |  |  |  |
| IL-17A, pg/mL | 597 | 4.29 (6.88) | 2.7 (6.11) | 0.89 | 62.98 | | 1.03 (1.89) | |  |  |  |  |
|  |  |  |  |  |  | |  | |  |  |  |  |
| **Autophagy/Oxidative stress** |  |  |  |  | | | |  | | |  | |
| P62, ng/mL | 628 | 0.99 (5.1) | 0.33 (0.7) | 0.1 | 69.59 | | -2.05 (1.97) | |  |  |  |  |
| Beclin-1, ng/mL | 661 | 0.56 (0.88) | 0.46 (0.37) | 0.1 | 87.29 | | -1.05 (0.61) | |  |  |  |  |
| SIRT1, pg/mL | 628 | 2484.94 (2173.69) | 2011.2 (2575.92) | 0.032 | 98.41 | | 10.96 (1.84) | |  |  |  |  |
| ATG5, ng/mL | 608 | 24.58 (44.35) | 16.85 (12.93) | 0.188 | 100 | | 4.10 (1.03) | |  |  |  |  |
|  |  |  |  |  |  | |  | |  |  |  |  |
| **ECM-turnover** |  |  |  |  | |  | | | |  | |  |
| MMP-9, ng/mL | 611 | 239.74 (596.03) | 143.9 (156.8) | 0.188 | 100 | | 7.17 (1.30) | |  |  |  |  |
| TGF-ß1, pg/mL | 611 | 18.97 (14.01) | 15.08 (17.97) | 0.019 | 100 | | 3.84 (1.12) | |  |  |  |  |
| PDGF-AA, pg/mL | 597 | 2831.27 (2377.38) | 2079.17 (2871.8) | 10.2 | 100 | | 10.91 (1.41) | |  |  |  |  |
| EGF, pg/mL | 597 | 113.01 (163.45) | 50.57 (113.84) | 3.13 | 96.98 | | 5.68 (1.93) | |  |  |  |  |
| VEGF-A, pg/mL | 597 | 173.3 (243.59) | 84.14 (207.43) | 0.91 | 91.79 | | 5.70 (2.94) | |  |  |  |  |

Abbreviations: SD—standard deviation, IQR—interquartile range, LOD—level of detection, log2— data after log2 transformation IL-1β—interleukin 1β, IL-8—interleukin 8, TNFα—tumor necrosis factor α, IFN-γ—interferon γ, p62—protein sequestosome 1, SIRT1—sirtuin 1, ATG5- autophagy protein 5, MMP-9—matrix metalloproteinase 9, PDGF-AA—platelet-derived growth factor AA, TGF-β1—transforming growth factor β1, IL-4—interleukin 4, IL-13—interleukin 13, IL-17A—interleukin 17A, EGF—epidermal growth factor, VEGF-A—vascular endothelial growth factor A; pg/mL—picograms per millilitre, ng/mL—nanograms per millilitre.

Table S4: Cord blood protein levels in infants born to mothers with asthma during pregnancy.

|  |  | **Adjusted models** | | | |
| --- | --- | --- | --- | --- | --- |
| Proteins |  | **β-coef** | **95% CI** | **p-value** | **p_adj_-value** |
| **Inflammatory associated** | | |  |  |  |
| IFN-γ |  | -0.77 | -1.21 – -0.32 | 0.001 | **0.001** |
| IL-1β |  | 0.13 | -0.74 – 1.00 | 0.776 | 0.831 |
| IL-8 |  | 0.37 | -0.44 – 1.17 | 0.372 | 0.465 |
| TNF-α |  | 0.21 | -0.04 – 0.45 | 0.09 | 0.150 |
| IL-4 |  | -0.65 | -1.28 – -0.03 | 0.04 | 0.150 |
| IL-13 |  | -0.67 | -0.65 - -146 | 0.09 | 0.100 |
| IL-17A |  | -0.96 | -1.81 - -0.10 | 0.03 | 0.090 |
| **Autophagy/Oxidative stress** | | |  |  |  |
| p62 |  | 1.15 | 0.30 – 2.00 | 0.01 | **0.037** |
| Beclin-1 |  | -0.31 | -0.53 – -0.10 | 0.001 | **0.005** |
| SIRT1 |  | -0.25 | -0.88 – 0.37 | 0.426 | 0.491 |
| ATG5 |  | -0.04 | -0.42 – 0.34 | 0.845 | 0.845 |
| **ECM-turnover** | | |  |  |  |
| MMP-9 |  | -0.67 | -1.07 – -0.27 | 0.001 | **0.005** |
| PDGF-AA |  | -0.50 | -1.01 – -0.01 | 0.06 | 0.128 |
| EGF |  | -0.51 | -1.26 – 0.24 | 0.182 |  |
| VEGF-A |  | -0.88 | -2.00 – 0.24 | 0.127 | 0.190 |
| TGF-ß1 |  | -0.23 | -0.66 – 0.17 | 0.256 | 0.349 |

Abbreviations: β-coef—beta-coefficient; 95% CI—95% confidence interval; Adj.—adjusted; ECM-turnover—extracellular matrix turnover; IFN-γ—interferon gamma; p62—ubiquitin-binding protein sequestosome 1; MMP-9—matrix metalloproteinase 9; PDGF-AA—platelet-derived growth factor; IL-4—interleukin 4; Each row represents results from a Tobit regression model, where maternal asthma during pregnancy is the primary exposure. Estimates are reported as coefficients with 95% CI log2-transformed protein levels, adjusted for sex, having siblings at birth, gestational age, maternal smoking, mode of delivery, birth weight in z-score, study center, and time of cord blood processing. P and p_adj_-values <0.05 (adjusted using the Benjamini–Hochberg method) are shown in bold.

Table S5: Demographic data of subset of infants with lung function available by grouping: infants born to non-asthmatic mothers versus infants born to asthmatic mothers

|  | **Born to asthmatic mothers**  **n=75** | **Born to**  **non-asthmatic mothers**  **n=420** | **Total**  **(n=495)** | **p-value** |
| --- | --- | --- | --- | --- |
| **Maternal baseline characteristics** |  |  |  |  |
| Maternal smoking during pregnancy *n (%)* | 6 (8.0%) | 26 (6.2%) | 32 (6.5%) | 0.608 |
| **Infant baseline characteristics** |  |  |  |  |
| First born *n (%)* | 39 (52.0%) | 190 (45.2%) | 229 (46.3%) | 0.315 |
| Male *n (%)* | 38 (50.7%) | 212 (50.5%) | 250 (50.5%) | 1.00 |
| Delivery type: |  |  |  | **0.042** |
| Vaginal *n (%)* | 52 (70.3%) | 340 (81.2%) | 392 (79.5%) |  |
| Cesarean section *n (%)* | 22 (29.7%) | 79 (18.8%) | 101 (20.5%) |  |
| Season of birth: |  |  |  | **0.033** |
| Winter *n (%)* | 22 (29.3%) | 62 (14.8%) | 84 (17.0%) |  |
| Spring *n (%)* | 20 (26.7%) | 132 (31.4%) | 152(30.7%) |  |
| Summer *n (%)* | 15 (20.0%) | 109 (26.0%) | 124 (23.5%) |  |
| Fall *n (%)* | 18 (24.0%) | 117 (27.9%) | 135 (27.3%) |  |
| Gestational age in weeks † | 39.6 (1.2) | 39.8 (1.1) | 39.7 (1.1) | 0.181 |
| Weight at birth, kg | 3.48 (0.51) | 3.39 (0.44) | 3.40 (0.45) | 0.108 |
| Weight at birth, z-score | 0.07 (1.00) | -0.19 (0.85) | -0.15 (0.88) | **0.020** |
| Length at birth, cm | 51.1 (2.87) | 49.5 (2.04) | 49.8 (2.25) | **<0.001** |
| Gestational age at test | 45.9 (1.65) | 44.9 (1.16) | 45.0 (1.29) | **<0.001** |
| Weight at test, kg | 4.70 (0.70) | 4.40 (0.54) | 4.45 (0.58) | **<0.001** |
| Length at test, cm | 55.8 (2.76) | 54.6 (2.46) | 54.8 (2.54) | **0.001** |
| Breastfeeding up to test date | 56 (74.7%) | 404 (97.1%) | 460 (93.7%) | **<0.001** |

Categoric variables are presented as counts and percentages; continuous variables are presented as means with SDs. Groups were compared using either a t-test or a chi-square, as appropriate. P-values <0.05 are shown in bold.

Table S6: Protein markers and their association with lung function at 4- 6 weeks of age

|  | |  | **Adjusted models** | | | | | | | |  |
| --- | --- | --- | --- | --- | --- | --- | --- | --- | --- | --- | --- |
| **Lung function** | |  | β-coef | | **95% CI** | | **p-value** | | **p_adj_-value** | |  |
| **Inflammation/Senescence** | | | |  | |  | |  | |  | |
| **IFN-γ** |  | | |  | |  | |  | |  | |
| tPTEF/tE% | |  | -0.29 | | -1.22 – 0.64 | | 0.545 | | 0.545 | |  |
| Minute ventilation | |  | 1.49 | | -20.06 – 23.03 | | 0.892 | | 0.743 | |  |
| FRC | |  | -1.26 | | -2.41 – -0.11 | | **0.032** | | 0.063 | |  |
| LCI | |  | 0.03 | | -0.03 – 0.09 | | 0.286 | | 0.518 | |  |
| **Autophagy/Oxidative stress** | | | | |  | |  | |  | |  |
| **p62** | |  |  | |  | |  | |  | |  |
| tPTEF/tE% | |  | -1.37 | | -2.72 – 0.04 | | **0.036** | | 0.108 | |  |
| Minute ventilation | |  | -16.18 | | -28.44 – -3.91 | | **0.009** | | **0.032** | |  |
| FRC | |  | -0.05 | | -0.68 – 0.59 | | 0.883 | | 0.883 | |  |
| LCI | |  | -0.01 | | -0.09 – 0.06 | | 0.571 | | 0.456 | |  |
| **ECM-turnover** | |  |  | |  | |  | |  | |  |
| **MMP-9** | |  |  | |  | |  | |  | |  |
| tPTEF/tE% | |  | 0.53 | | -0.49 – 1.55 | | 0.309 | | 0.517 | |  |
| Minute ventilation | |  | 7.94 | | -14.90 – 30.79 | | 0.494 | | 0.921 | |  |
| FRC | |  | -1.27 | | -2.53 – -0.01 | | **0.048** | | 0.063 | |  |
| LCI | |  | 0.07 | | 0.02 – 0.14 | | **0.011** | | **0.036** | |  |

Abbreviations: β-Coef—coefficient; CI—confidence interval; Adj—adjusted; ECM-turnover—extracellular matrix turnover; IFN-γ—interferon gamma; p62—ubiquitin-binding protein sequestosome 1; MMP-9—matrix metalloproteinase 9; tPTEF/tE%—the ratio of time to peak tidal expiratory flow as a percentage of total expiratory time; FRC—functional residual capacity; LCI—lung clearance index;
Each row shows results from linear regression models, where protein levels at birth were the primary exposure. Lung function estimates are reported as coefficients with 95% CI per 1-unit increase in log2-transformed protein levels. Adjusted for asthma during pregnancy, sex, having siblings at birth, postmenstrual age at lung function test in weeks, weight at test, maternal smoking during pregnancy, mode of delivery, study centre, and time of cord blood processing in days. P and p_adj_-values <0.05 adjusted using the Benjamini–Hochberg method) are shown in bold.

Table S7: Demographic characteristics according to bronchiolitis hospitalization

|  |  | **Bronchiolitis**  **n=22** | **Non-bronchiolitis**  **n=583** | **p-value** |
| --- | --- | --- | --- | --- |
| **Maternal baseline characteristics** |  |  |  |  |
| Maternal smoking during pregnancy *n (%)* |  | 4 (17.9%) | 45 (7.7%) | 0.094 |
| Maternal asthma during pregnancy *n (%)* |  | 5 (22.7%) | 123 (21.1%) | 0.794 |
| **Infant baseline Characteristics** |  |  |  |  |
| First born *n (%)* |  | 5 (22.7%) | 276 (47.3%) | **0.028** |
| Male *n (%)* |  | 16 (72.7%) | 290 (49.7%) | **0.049** |
| Delivery type: |  |  |  | 1.000 |
| Vaginal *n (%)* |  | 18 (81.8%) | 458 (79.1%) |  |
| Cesarean section *n (%)* |  | 4 (18.2%) | 121 (20.9%) |  |
| Season of birth: |  |  |  | 0.417 |
| Winter *n (%)* |  | 4 (18.2%) | 100 (17.2%) |  |
| Spring *n (%)* |  | 4 (18.2%) | 189 (32.4%) |  |
| Summer *n (%)* |  | 8 (36.4%) | 137 (23.5%) |  |
| Fall *n (%)* |  | 6 (27.3%) | 157 (26.9%) |  |
| Gestational age in weeks † |  | 39.6 (1.3) | 39.7 (1.1) | 0.729 |
| Weight at birth, kg |  | 3.42 (0.51) | 3.41 (0.45) | 0.965 |
| Weight at birth, z-score |  | -0.16 (0.91) | -0.12 (0.8) | 0.856 |
| Length at birth, cm |  | 49.5 (3.3) | 49.9 (2.3) | 0.431 |

Categoric variables are presented as counts and percentages; continuous variables are presented as means with standard deviation (SDs) and † and as median with interquartile range (IQR). Groups were compared using either a t-test or a chi-square, as appropriate. P-values <0.05 are shown in bold.

Table S8: Demographic characteristics according to asthma diagnosis in childhood

|  |  | **Asthma in childhood**  **n=29** | **Non-asthma**  **in childhood**  **n=392** | **p-value** |
| --- | --- | --- | --- | --- |
| **Maternal baseline characteristics** |  |  |  |  |
| Maternal smoking during pregnancy *n (%)* |  | 3 (10.3%) | 24 (6.1%) | 0.370 |
| Maternal asthma during pregnancy *n (%)* |  | 16 (55.2%) | 35 (8.9%) | **<0.001** |
| **Infant baseline Characteristics** |  |  |  |  |
| First born *n (%)* |  | 9 (31.0%) | 186 (47.5%) | 0.087 |
| Male *n (%)* |  | 19 (65.5%) | 186 (47.5%) | 0.060 |
| Delivery type: |  |  |  | 0.496 |
| Vaginal *n (%)* |  | 25 (86.2%) | 318 (81.1%) |  |
| Cesarean section *n (%)* |  | 4 (13.8%) | 74 (18.9%) |  |
| Season of birth: |  |  |  | 0.303 |
| Winter *n (%)* |  | 7 (24.1%) | 63 (16.1%) |  |
| Spring *n (%)* |  | 9 (31.0%) | 127 (32.4%) |  |
| Summer *n (%)* |  | 9 (31.0%) | 93 (23.7%) |  |
| Fall *n (%)* |  | 4 (13.8%) | 109 (13.8%) |  |
| Gestational age in weeks † |  | 39.5 (1.4) | 39.8 (1.2) | 0.209 |
| Weight at birth, kg |  | 3.59 (0.51) | 3.39 (0.44) | **0.020** |
| Weight at birth, z-score |  | 0.30 (0.91) | -0.17 (0.9) | **0.005** |
| Length at birth, cm |  | 56.1 (1.90) | 54.6 (2.27) | **0.002** |

Categoric variables are presented as counts and percentages; continuous variables are presented as means with standard deviation (SDs) and † and as median with interquartile range (IQR). Groups were compared using either a t-test or a chi-square, as appropriate. P-values <0.05 are shown in bold.

Table S9: Sensitivity analysis excluding infants born by Cesarean section. Results are presented according to model for each aim based on three main markers

| Proteins |  | **β-Coef** | **95% CI** | **p-value** |
| --- | --- | --- | --- | --- |
| **IFN-γ** | | |  |  |
| Tobit regression model^a^ |  | -1.20 | -1.71 – -0.70 | **<0.0001** |
| Lung function model ^b^ |  |  |  |  |
| Minute ventilation |  | -3.96 | -30.65 – 22.73 | 0.771 |
| tPTEF/tE% |  | -0.03 | -1.10 – 1.03 | 0.950 |
| FRC |  | -1.53 | -2.86 – -0.20 | **0.023** |
| LCI |  | 0.14 | -4.83 – 0.08 | 0.450 |
| Bronchiolitis model ^c^ |  |  |  |  |
| **p62** | | |  |  |
| Tobit regression model ^a^ |  | 1.04 | -0.05 – 2.13 | 0.060 |
| Lung function model ^b^ |  |  |  |  |
| Minute ventilation |  | -20.21 | -5.87 – -2.77 | **0.006** |
| tPTEF/tE% |  | -0.78 | -0.22 – -2.74 | **0.006** |
| FRC |  | -0.26 | -0.97 – 0.43 | 0.459 |
| LCI |  | 0.01 | -0.04 – 0.02 | 0.941 |
| Bronchiolitis model ^c^ |  | aOR 1.25 | 1.02 – 1.52 | **0.030** |
| **MMP-9** | | |  |  |
| Tobit regression model ^a^ |  | -0.69 | -1.18 – -0.19 | **0.006** |
| Lung function model ^b^ |  |  |  |  |
| Minute ventilation |  | 1.52 | -25.97 – 29.02 | 0.913 |
| tPTEF/tE% |  | 0.92 | -0.18 – 2.02 | 0.100 |
| FRC |  | -1.31 | -2.72 – 0.09 | 0.067 |
| LCI |  | 0.06 | -3.48 – 0.13 | 0.063 |
| Bronchiolitis model ^c^ |  | aOR 0.48 | 0.29 – 0.83 | **0.009** |

Abbreviations: Estimates are reported according to models for ^a^ Tobit regression (first aim, part one), ^b^ Lung function (second aim, part one), ^c^ Bronchiolitis hospitalization (second aim, part two), Coef—coefficient; CI—confidence interval;; aOR—adjusted odds ratio; IFN-γ—interferon gamma; p62—ubiquitin-binding protein sequestosome 1; MMP-9—matrix metalloproteinase 9; tPTEF/tE%—the ratio of time to peak tidal expiratory flow as a percentage of total expiratory time; FRC—functional residual capacity; LCI—lung clearance index.

Table S10: Tobit regression model results showing IFN-γ levels in infants born to mothers with asthma during pregnancy as the main exposure and adjusted for all covariates

| Regression Results for IFN-γ | | | | |
| --- | --- | --- | --- | --- |
| Term | Estimate | 95% CI | | p-value |
| (Intercept):1 | 3.95 | 0.19 | 7.71 | 0.040 |
| (Intercept):2 | 0.15 | 0.09 | 0.21 | 0.000 |
| **Asthma during pregnancy** | **-0.77** | **-1.21** | **-0.32** | **0.001** |
| Male sex | 0.20 | 0.00 | 0.40 | 0.053 |
| Having siblings | -0.18 | -0.39 | 0.03 | 0.099 |
| Gestational age, weeks | 0.01 | -0.08 | 0.10 | 0.808 |
| Smoker during pregnancy | -0.25 | -0.62 | 0.12 | 0.192 |
| Cesarean section delivery | -0.49 | -0.74 | -0.23 | 0.000 |
| Birth weight, z-Score | -0.06 | -0.18 | 0.07 | 0.368 |
| Study center | 0.17 | 0.02 | 0.32 | 0.025 |
| Sample processing time, days | 0.02 | -0.12 | 0.15 | 0.798 |

Abbreviations: IFN-γ—interferon gamma.

Table S11: Tobit regression model results showing p62 levels in infants born to mothers with asthma during pregnancy as the main exposure and adjusted for all covariates

| Regression Results for p62 | | | | |
| --- | --- | --- | --- | --- |
| Term | Estimate | 95% CI | | p-value |
| (Intercept):1 | -5.80 | -13.35 | 1.74 | 0.132 |
| (Intercept):2 | 0.81 | 0.74 | 0.89 | 0.000 |
| **Asthma during pregnancy** | **1.15** | **0.30** | **2.00** | **0.008** |
| Male sex | 0.35 | -0.05 | 0.75 | 0.088 |
| Having siblings | -0.53 | -0.96 | -0.11 | 0.014 |
| Gestational age, weeks | 0.05 | -0.13 | 0.24 | 0.567 |
| Smoker during pregnancy | -0.32 | -1.20 | 0.55 | 0.468 |
| Cesarean section delivery | 0.01 | -0.50 | 0.53 | 0.955 |
| Birth weight, z-Score | 0.15 | -0.09 | 0.39 | 0.226 |
| Study center | 0.15 | -0.14 | 0.44 | 0.300 |
| Sample processing time, days | 0.68 | 0.41 | 0.95 | 0.000 |

Abbreviations: p62—ubiquitin-binding protein sequestosome 1.

Table S12: Tobit regression model results showing MMP-9 levels in infants born to mothers with asthma during pregnancy as the main exposure and adjusted for all covariates

| Regression Results for MMP-9 | | | | |
| --- | --- | --- | --- | --- |
| Term | Estimate | 95% CI | | p-value |
| (Intercept):1 | 0.61 | -2.76 | 3.98 | 0.723 |
| (Intercept):2 | 0.06 | 0.00 | 0.12 | 0.049 |
| **Asthma during pregnancy** | **-0.67** | **-1.07** | **-0.27** | **0.001** |
| Male sex | 0.09 | -0.09 | 0.27 | 0.328 |
| Having siblings | -0.11 | -0.30 | 0.08 | 0.270 |
| Gestational age, weeks | 0.16 | 0.08 | 0.25 | 0.000 |
| Smoker during pregnancy | -0.04 | -0.38 | 0.29 | 0.806 |
| Cesarean section delivery | -0.48 | -0.70 | -0.25 | 0.000 |
| Birth weight, z-score | 0.03 | -0.08 | 0.14 | 0.630 |
| Study center | -0.02 | -0.15 | 0.12 | 0.795 |
| Sample processing time, days | 0.42 | 0.30 | 0.55 | 0.000 |

Abbreviations: MMP-9—matrix metalloproteinase 9;

Table S13: Cord blood protein levels in infants born to mothers with asthma during pregnancy in sensitivity models adjusting according to LASSO.

|  |  | **Adjusted models** | | | |
| --- | --- | --- | --- | --- | --- |
| Proteins |  | **β-coef** | **95% CI** | **p-value** | **p_adj_-value** |
| **Inflammatory associated** | | |  |  |  |
| IFN-γ |  | **-0.77** | **-1.21 – -0.32** | **0.001** | **0.004** |
| IL-1β |  | 0.22 | -0.65 – 1.09 | 0.615 | 0.702 |
| IL-8 |  | 0.37 | -0.44 – 1.17 | 0.372 | 0.541 |
| TNF-α |  | 0.20 | -0.06 – 0.47 | 0.124 | 0.198 |
| IL-4 |  | **-0.65** | **-1.28 – -0.03** | **0.040** | 0.091 |
| IL-13 |  | **-0.45** | **-0.89 – -0.005** | **0.047** | 0.094 |
| IL-17A |  | **-0.95** | **-1.49 - -0.40** | **0.001** | 0.004 |
| **Autophagy/Oxidative stress** | | |  |  |  |
| p62 |  | **0.80** | **0.20 – 1.39** | **0.008** | **0.020** |
| Beclin-1 |  | **-0.31** | **-0.53 – -0.10** | **0.001** | **0.004** |
| SIRT1 |  | -0.01 | -0.46 – 0.42 | 0.946 | 0.946 |
| ATG5 |  | -0.02 | -0.39 – 0.35 | 0.918 | 0.946 |
| **ECM-turnover** | | |  |  |  |
| MMP-9 |  | **-0.61** | **-0.86 – -0.36** | **<0.001** | **<0.001** |
| PDGF-AA |  | -0.50 | -1.01 – -0.01 | 0.056 | 0.099 |
| EGF |  | -0.13 | -0.52 – 0.26 | 0.510 | 0.657 |
| VEGF-A |  | **-0.95** | **-1.59 – -0.32** | **0.003** | **0.009** |
| TGF-ß1 |  | -0.08 | -0.33 – 0.17 | 0.534 | 0.657 |

Abbreviations: LASSO—least absolute shrinkage and selection operator; β-coef—beta-coefficient; 95% CI—95% confidence interval; Adj.—adjusted; ECM-turnover—extracellular matrix turnover; IFN-γ—interferon gamma; p62—ubiquitin-binding protein sequestosome 1; MMP-9—matrix metalloproteinase 9; PDGF-AA—platelet-derived growth factor; IL-4—interleukin 4; Each row represents results from a Tobit regression model, where maternal asthma during pregnancy is the primary exposure. Estimates are reported as coefficients with 95% CI log2-transformed protein levels, adjusted for variables chosen by LASSO from this list: sex, having siblings at birth, gestational age, maternal smoking, mode of delivery, birth weight in z-score, study center, and time of cord blood processing. P and p_adj_-values <0.05 (adjusted using the Benjamini–Hochberg method) are shown in bold.

**Supplementary Figures**

Figure S1: Flow diagram illustrating the study population deriving from both cohort studies


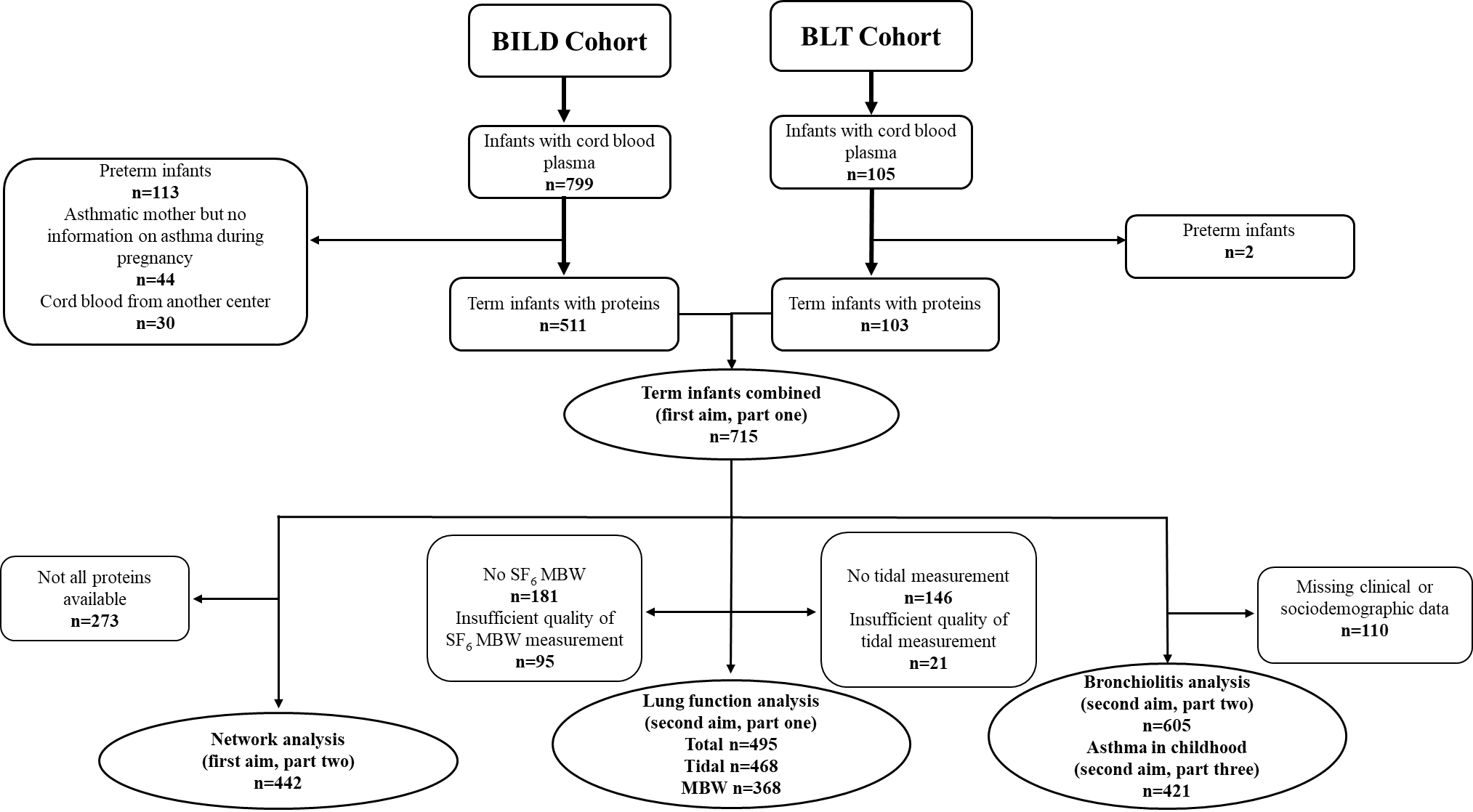


Abbreviations: BILD—Basel-Bern Infant Lung Development; BLT—Breathing for Life Trial; SF6 MBW—sulfur hexafluoride 6 multiple breath washout.

Figure S2: Comparison of protein levels in infants born to mothers with asthma during pregnancy versus those born to non-asthmatic mothers.
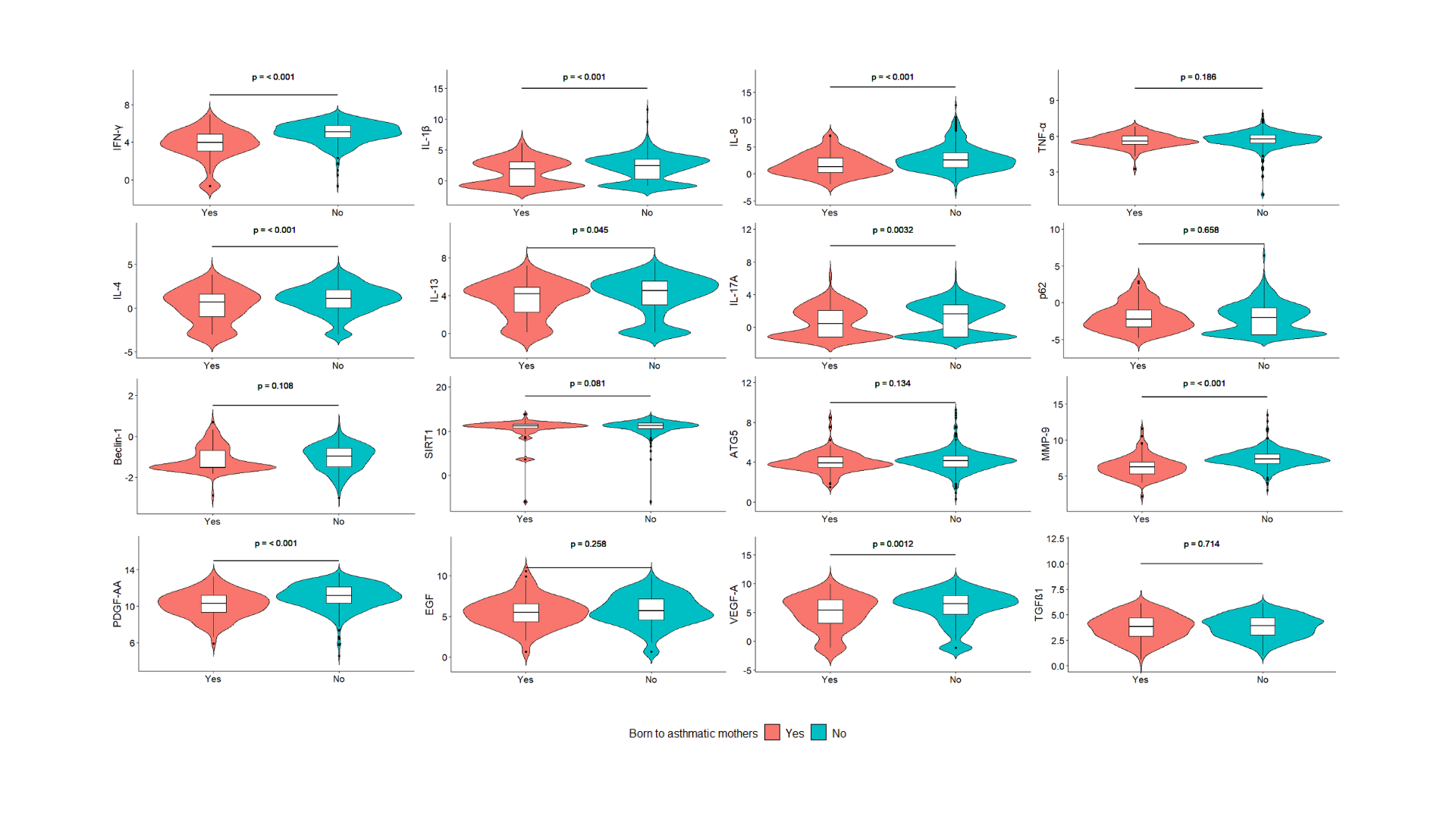
Abbreviations: IL-1β—interleukin 1β, IL-4—interleukin4, IL-8—interleukin 8, TNFα—tumor necrosis factor α, IFN-γ—interferon gamma, MMP-9—matrix metalloproteinase 9, PDGF-AA—platelet-derived growth factor AA, IL-13—interleukin 13, IL-17A—interleukin 17A, EGF—epidermal growth factor, VEGF-A—vascular endothelial growth factor A. Groups were compared using an independent *t*-test to assess differences in the mean values of each variable between groups. P-values were calculated for each comparison with values <0.05 considered statistically significant. Only showing significant differences.

Figure S3: Spearman’s Correlation of Cord Blood Protein Levels in Infants Born to Asthmatic and Non-Asthmatic Mothers according to correlation network analysis.


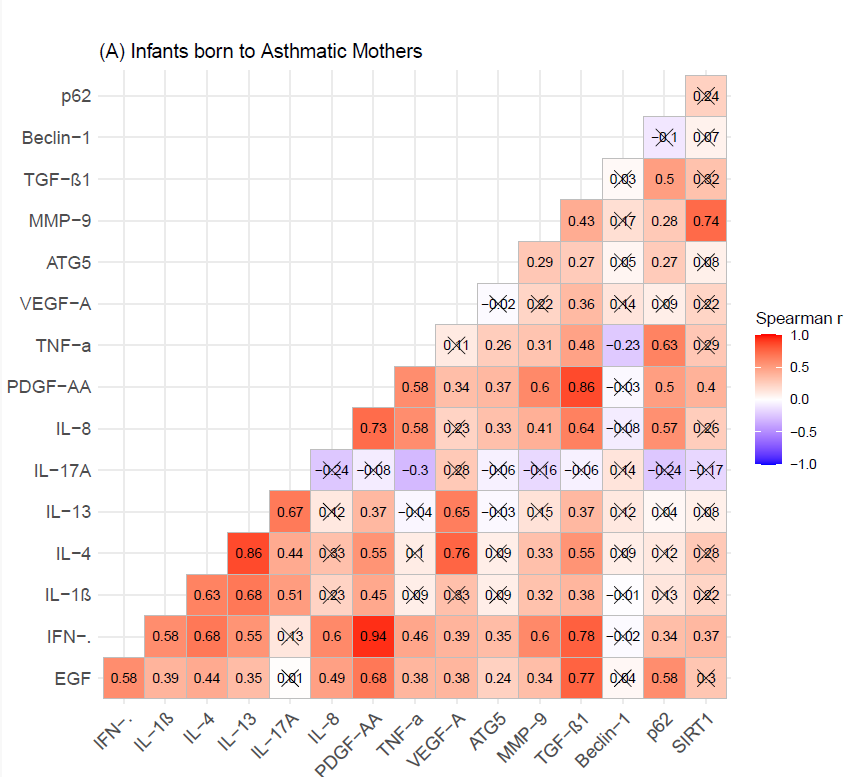


*
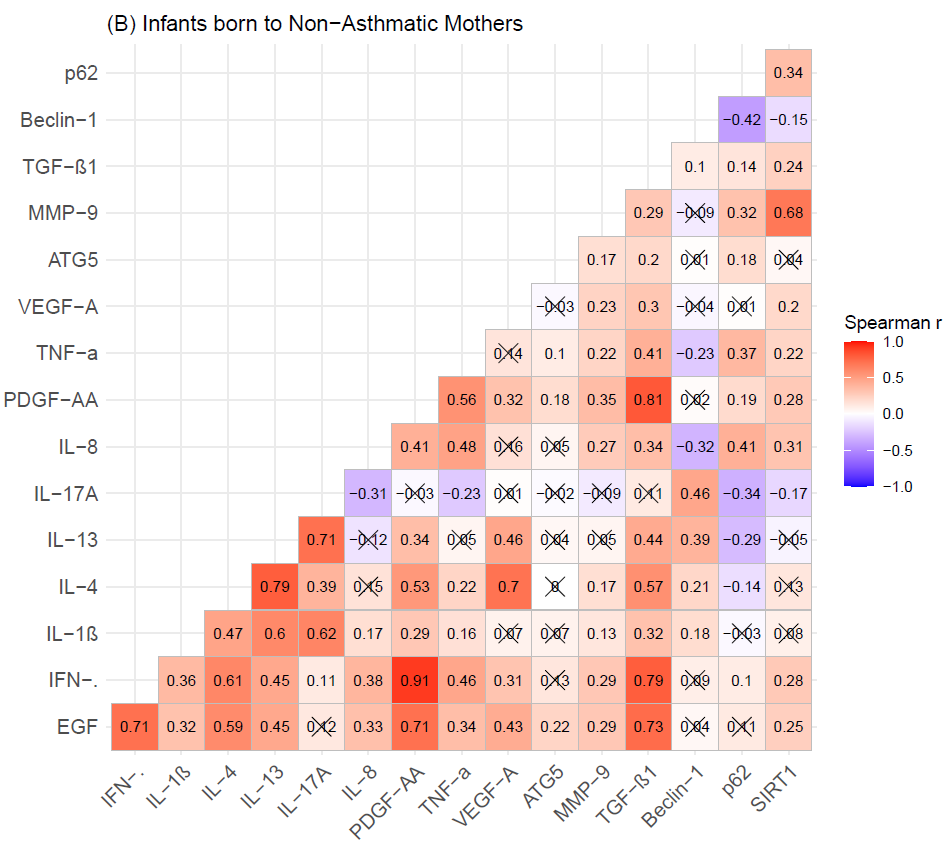
*

Spearman’s correlation heatmap illustrating the associations between protein levels in cord blood from infants born to asthmatic mothers (A) and infants born to non-asthmatic mothers (B). Each square represents the Spearman’s correlation coefficient (r) between a given protein and another. The color gradient indicates the strength and direction of the correlation, ranging from blue (strong negative correlation) to red (strong positive correlation). Squares marked with an “×” denote correlations that did not reach statistical significance (p ≥ 0.05). Abbreviations: IL-1β—interleukin 1β, IL-4—interleukin4, IL-8—interleukin 8, TNFα—tumor necrosis factor α, IFN-γ—interferon gamma, MMP-9—matrix metalloproteinase 9, PDGF-AA—platelet-derived growth factor AA, IL-13—interleukin 13, IL-17A—interleukin 17A, EGF—epidermal growth factor, VEGF-A—vascular endothelial growth factor A.

**References**

1. Murphy VE, Jensen ME, Mattes J, Hensley MJ, Giles WB, Peek MJ, et al. The Breathing for Life Trial: a randomised controlled trial of fractional exhaled nitric oxide (FENO)-based management of asthma during pregnancy and its impact on perinatal outcomes and infant and childhood respiratory health. BMC Pregnancy and Childbirth. 2016;16(1):111.

2. Antoshina DV, Balandin SV, Bogdanov IV, Vershinina MA, Sheremeteva EV, Toropygin IY, et al. Antimicrobial Activity and Immunomodulatory Properties of Acidocin A, the Pediocin-like Bacteriocin with the Non-Canonical Structure. Membranes (Basel). 2022;12(12).

3. Lee SW, Park SE, Jeong GS. Sporadic cell death in macroscale 3D tumor grafts with high drug resistance by activating cell-ECM interactions. Biofabrication. 2021;13(4):045022.

4. Paunovic V, Vucicevic L, Misirkic Marjanovic M, Perovic V, Ristic B, Bosnjak M, et al. Autophagy Receptor p62 Regulates SARS-CoV-2-Induced Inflammation in COVID-19. Cells. 2023;12(9).

5. Klionsky DJ, Abdel-Aziz AK, Abdelfatah S, Abdellatif M, Abdoli A, Abel S, et al. Guidelines for the use and interpretation of assays for monitoring autophagy (4th edition)(1). Autophagy. 2021;17(1):1-382.

6. Viana-Mattioli S, Nunes P, Cavalli R, Sandrim V. Analysis of SIRT1 Expression in Plasma and in an In Vitro Model of Preeclampsia. Oxid Med Cell Longev. 2020;2020:4561083.

7. Künstle N, Gorlanova O, Marten A, Müller L, Sharma P, Röösli M, et al. Differences in autophagy marker levels at birth in preterm vs. term infants. Pediatric Research. 2024.

8. Bates JH, Schmalisch G, Filbrun D, Stocks J. Tidal breath analysis for infant pulmonary function testing. ERS/ATS Task Force on Standards for Infant Respiratory Function Testing. European Respiratory Society/American Thoracic Society. Eur Respir J. 2000;16(6):1180-92.

9. Frey U, Stocks J, Coates A, Sly P, Bates J. Specifications for equipment used for infant pulmonary function testing. ERS/ATS Task Force on Standards for Infant Respiratory Function Testing. European Respiratory Society/ American Thoracic Society. Eur Respir J. 2000;16(4):731-40.

10. Fuchs O, Latzin P, Thamrin C, Stern G, Frischknecht P, Singer F, et al. Normative data for lung function and exhaled nitric oxide in unsedated healthy infants. European Respiratory Journal. 2011;37(5):1208.

11. Latzin P, Kuehni CE, Baldwin DN, Roiha HL, Casaulta C, Frey U. Elevated exhaled nitric oxide in newborns of atopic mothers precedes respiratory symptoms. Am J Respir Crit Care Med. 2006;174(12):1292-8.

12. Johnson WE, Li C, Rabinovic A. Adjusting batch effects in microarray expression data using empirical Bayes methods. Biostatistics. 2007;8(1):118-27.

13. Barnes JL, Yoshida M, He P, Worlock KB, Lindeboom RGH, Suo C, et al. Early human lung immune cell development and its role in epithelial cell fate. Sci Immunol. 2023;8(90):eadf9988.

14. Ikegami T, Tsuda A, Karube A, Kodama H, Hirano H, Tanaka T. Effects of intrauterine IL-6 and IL-8 on the expression of surfactant apoprotein mRNAs in the fetal rat lung. European Journal of Obstetrics & Gynecology and Reproductive Biology. 2000;93(1):97-103.

15. You K, Gu H, Yuan Z, Xu X. Tumor Necrosis Factor Alpha Signaling and Organogenesis. Frontiers in Cell and Developmental Biology. 2021;9.

16. Ballard PL, Liley HG, Gonzales LW, Odom MW, Ammann AJ, Benson B, et al. Interferon-gamma and Synthesis of Surfactant Components by Cultured Human Fetal Lung. American Journal of Respiratory Cell and Molecular Biology. 1990;2(2):137-43.

17. Loering S, Cameron GJM, Starkey MR, Hansbro PM. Lung development and emerging roles for type 2 immunity. The Journal of Pathology. 2019;247(5):686-96.

18. Bjørkøy G, Lamark T, Brech A, Outzen H, Perander M, Overvatn A, et al. p62/SQSTM1 forms protein aggregates degraded by autophagy and has a protective effect on huntingtin-induced cell death. J Cell Biol. 2005;171(4):603-14.

19. Yeganeh B, Lee J, Ermini L, Lok I, Ackerley C, Post M. Autophagy is required for lung development and morphogenesis. J Clin Invest. 2019;129(7):2904-19.

20. Greenlee KJ, Werb Z, Kheradmand F. Matrix metalloproteinases in lung: multiple, multifarious, and multifaceted. Physiol Rev. 2007;87(1):69-98.

21. Lindahl P, Karlsson L, Hellström M, Gebre-Medhin S, Willetts K, Heath JK, et al. Alveogenesis failure in PDGF-A-deficient mice is coupled to lack of distal spreading of alveolar smooth muscle cell progenitors during lung development. Development. 1997;124(20):3943-53.

22. Kumar VH, Ryan RM. Growth factors in the fetal and neonatal lung. Front Biosci. 2004;9:464-80.
